# The Microbiome of the Medicinal Plants *Achillea millefolium* L. and *Hamamelis virginiana* L.

**DOI:** 10.3389/fmicb.2021.696398

**Published:** 2021-07-20

**Authors:** Simon Sauer, Leon Dlugosch, Dietmar R. Kammerer, Florian C. Stintzing, Meinhard Simon

**Affiliations:** ^1^WALA Heilmittel GmbH, Bad Boll, Germany; ^2^Institute for Chemistry and Biology of the Marine Environment, University of Oldenburg, Oldenburg, Germany

**Keywords:** fungi, bacteria, epiphytic, endophytic, diversity, phyllosphere, season, core-community

## Abstract

In the recent past many studies investigated the microbiome of plants including several medicinal plants (MP). Microbial communities of the associated soil, rhizosphere and the above-ground organs were included, but there is still limited information on their seasonal development, and in particular simultaneous investigations of different plant organs are lacking. Many studies predominantly addressed either the prokaryotic or fungal microbiome. A distinction of epi- and endophytic communities of above-ground plant organs has rarely been made. Therefore, we conducted a comprehensive investigation of the bacterial and fungal microbiome of the MP *Achillea millefolium* and studied the epi- and endophytic microbial communities of leaves, flower buds and flowers between spring and summer together with the microbiome of the associated soil at one location. Further, we assessed the core microbiome of *Achillea* from four different locations at distances up to 250 km in southern Germany and Switzerland. In addition, the bacterial and fungal epi- and endophytic leaf microbiome of the arborescent shrub *Hamamelis virginiana* and the associated soil was investigated at one location. The results show a generally decreasing diversity of both microbial communities from soil to flower of *Achillea*. The diversity of the bacterial and fungal endophytic leaf communities of *Achillea* increased from April to July, whereas that of the epiphytic leaf communities decreased. In contrast, the diversity of the fungal communities of both leaf compartments and that of epiphytic bacteria of *Hamamelis* increased over time indicating plant-specific differences in the temporal development of microbial communities. Both MPs exhibited distinct microbial communities with plant-specific but also common taxa. The core taxa of *Achillea* constituted a lower fraction of the total number of taxa than of the total abundance of taxa. The results of our study provide a basis to link interactions of the microbiome with their host plant in relation to the production of bioactive compounds.

## Introduction

Epi- and endophytic microorganisms, including prokaryotes and fungi, colonize plants above ground (phyllosphere) and below ground (rhizosphere) and exhibit different ecological interactions with their host from beneficial and commensal symbiosis to pathogenic relationships (Hardoim et al., 2015; Jia et al., 2016; Brader et al., 2017). Uniquely shaped microbiomes were found in a variety of plants and their different compartments such as rhizosphere, leaf, flower, fruit, and seed (Copeland et al., 2015; Grudzinska-Sterno et al., 2016; Hamonts et al., 2018; Wei and Ashman, 2018; Grady et al., 2019; Comeau et al., 2020; Massoni et al., 2020). Embedded in a wide range of environmental constraints, the structure of the plant microbiome is far from random; its formation and temporal development is a continuous process, expressed by specific co-occurrence patterns and microbial interactions (Cardinale et al., 2015; Berg et al., 2016; Grady et al., 2019). Variables affecting the microbiome and its temporal development in the different compartments include environmental factors such as wind, rain, temperature and soil chemistry, leading to horizontal transmission of microbes and structuring mainly the epiphytic microbial communities (Müller et al., 2016). Some microbes can also establish in the phyllosphere by vertical transmission from seeds and plant compartments and affect mainly the endophytic microbial communities (Müller et al., 2016).

Past research on plant microbiomes has demonstrated growth-promoting effects (Naik et al., 2019), the enhancement of plant resistance against pathogens (Ansary et al., 2018; Vurukonda et al., 2018) and positive effects on overall plant fitness (Rodriguez et al., 2009; Uroz et al., 2019). Further, implications of the microbiome on agricultural management (Toju et al., 2018; Jones et al., 2019; Pacifico et al., 2019; Lyu et al., 2020), crop quality and postharvest processes (Rillig et al., 2018) have been addressed. Several studies investigated the microbiome of medicinal plants (MPs), e.g., *Origanum vulgare* (Pontonio et al., 2018; Castronovo et al., 2020), *Matricaria chamomilla, and Calendula officinalis* (Köberl et al., 2019) as well as *Salvia miltiorrhiza* (Chen et al., 2018; Huang et al., 2018). MPs are characterized by a high variety of unique secondary metabolites and many endophytes producing pharmacologically active substances (Köberl et al., 2013; Golinska et al., 2015; Ivanova et al., 2016; Rahman et al., 2017; Caruso et al., 2020; Gupta et al., 2020; Teimoori-Boghsani et al., 2020). Thus, beside the potential impact of the plant microbiome on pathogen resistance and general fitness evidence has accumulated that the microbiome has a significant impact on the production of secondary metabolites of MPs (Köberl et al., 2013; Schmidt et al., 2014; Jia et al., 2016; Huang et al., 2018; Pacifico et al., 2019). This has recently been specified for *Echinacea purpurea* (Maggini et al., 2017; Haron et al., 2019), *Camellia sinensis* (Sun et al., 2019), and *Cannabis sativa* (Taghinasab and Jabaji, 2020). Further work is still required to illuminate mutual interactions of MPs and their microbiome and its potential role in producing beneficial bioactive compounds.

A very important but almost unexplored aspect in this context is the seasonality of the microbiome. To obtain a high-quality herbal drug, the MP has to be harvested at the optimum developmental stage of the desired plant organ (Chen et al., 2012; Duckstein et al., 2012b; Duda et al., 2015). It is completely unclear how this optimum stage is reflected in the plant microbiome and how it establishes over time. As a first step toward this aim, Comeau et al. (2020) identified spatio-temporal variations in the microbiome of *C. sativa* at different developmental stages. Further, endophytic *Actinobacteria*, known for their high potential of producing bioactive compounds, vary seasonally in tropical MPs (Barman and Dkhar, 2020). Therefore, knowledge about the seasonal development and dynamics of the microbiome should be part of a comprehensive understanding of the complex interactions between MP metabolites and the plant’s microbiome. This may also have an impact on the pharmacological potential and further processing such as fermentation of MPs (Duckstein et al., 2012b; Hussain et al., 2016; Köberl et al., 2019).

To elucidate differences in microbial communities and their temporal dynamics of two MPs with different habitus and lifestyle we investigated the microbiome of *Achillea millefolium* (in the following *Achillea*) and *Hamamelis virginiana* (in the following *Hamamelis*). *Achillea*, commonly referred to as yarrow, is a perennial plant native to the Northern hemisphere of Asia, Europe and America. Pharmaceutical preparations of this MP are used to treat loss of appetite, gut complaints, wounds, and menstrual spasms (EMA/HMPC, 2018), to reduce inflammation (Benedek et al., 2007) and even show *in vitro* potential against cancer (Köngül et al., 2017; Pereira et al., 2018). *Hamamelis*, with the common name witch hazel, is a deciduous arborescent shrub or small tree native to eastern North America and is used in the treatment of inflammations of skin and mucous membranes, varicose veins, and hemorrhoids. Furthermore, *Hamamelis* is frequently applied in cosmetics, i.e., skin lotions, nourishing creams, pre-, and after-shaves (EMEA, 2009). The endophytic bacterial community of *Achillea* on hydrocarbon-contaminated and natural soil (Lumactud et al., 2016; Lumactud and Fulthorpe, 2018) and endophytic fungi of this plant have been investigated recently (Satari et al., 2016; Hatamzadeh et al., 2018, 2020). We extended the investigations of the microbiome of *Achillea* by including epi- and endophytic bacterial and fungal communities of the associated soil, leaf, flower bud and flower, including their temporal dynamics and a comparison of four different locations. We further investigated for the first time the epi- and endophytic microbiome of *Hamamelis*. We addressed the following questions: (i) how do the endo- and epiphytic bacterial and fungal communities of *Achillea* change from the early leaf development stage in spring to the flowering stage in summer and the respective microbial leaf communities of *Hamamelis* from spring to summer? (ii) How does the composition of the epi- and endophytic bacterial and fungal communities differ between each other and both plants? (iii) Does *Achillea* harbor a distinct core community of bacteria and fungi? Illumina Miseq sequencing was applied to generate amplicon datasets of the 16S rRNA gene of the bacterial and the intergenic transcribed spacer (ITS) for the fungal community. The results show that *Achillea* and *Hamamelis* exhibit distinct microbiomes above ground both in their bacterial and fungal communities, which also undergo seasonal changes and that *Achillea* harbors a specific core community when comparing different locations up to 250 km apart.

## Materials and Methods

### Sampling

Plant and soil samples were collected in 2016 during the growing period of both MPs in the biodynamic garden of WALA Heilmittel GmbH (location 1a) from April until July for *Achillea* and until September for *Hamamelis*, respectively, (Table 1). Both plants have been growing at this location in close proximity for more than a decade. To evaluate the impact of different locations, we sampled *Achillea* again in July 2018 at the same place (location 1b) in comparison with three other locations, i.e., wild plants beside a meadow with cows near Goeppingen (Germany; location 2), a meadow close to the river Thur near Kleinandelfingen (Switzerland; location 3) and in a biodynamic garden in the Swiss Alps (1,096 m above sea level; location 4).

**TABLE 1:** Sampling details of *Achillea millefolium* and *Hamamelis virginiana*, date, location, location ID, elevation above sea level (aSL), soil pH, and coordinates.

Species	Sampling date	Time series	Spatial comparison	Location	Location ID	Elevation (m aSL)	pH	Coordinates		Type
								N	E	
*Hamamelis*	06 Apr 2016	X		D- WALA garden	1a	444	n/a	48°37′45″	9°35′13″	Leaf, soil
*Hamamelis*	18 Apr 2016	X		D- WALA garden	1a	444	n/a	48°37′45″	9°35′13″	Leaf, soil
*Hamamelis*	04 May 2016	X		D- WALA garden	1a	444	n/a	48°37′45″	9°35′13″	Leaf, soil
*Hamamelis*	20 May 2016	X		D- WALA garden	1a	444	n/a	48°37′45″	9°35′13″	Leaf, soil
*Hamamelis*	04 Jul0.2016 *	X		D- WALA garden	1a	444	7.2	48°37′45″	9°35′13″	Leaf, soil
*Hamamelis*	20 Sep2016	X		D- WALA garden	1a	444	n/a	48°37′45″	9°35′13″	Leaf, soil
*Achillea*	11 Apr 2016	X		D- WALA garden	1a	444	n/a	48°37′45″	9°35′14″	Leaf, soil
*Achillea*	04 May 2016	X		D- WALA garden	1a	444	n/a	48°37′45″	9°35′14″	Leaf, soil
*Achillea*	10 Jun 2016	X		D- WALA garden	1a	444	n/a	48°37′45″	9°35′14″	Leaf, bud, soil
*Achillea*	23 Jul 2016 *	X		D- WALA garden	1a	444	7.7	48°37′45″	9°35′14″	Leaf, flower, soil
*Achillea*	19 Jul 2018		X	D- WALA garden	1b	444	7.7	48°37′45″	9°35′14″	Leaf, flower, soil
*Achillea*	12 Jul 2018		X	CH- Alp-garden	4	1096	7.6	46°43′41″	8°01′15″	Leaf, flower, soil
*Achillea*	12 Jul 2018		X	CH- Kleinandelfingen, meadow	3	378	7.8	47°35′54″	8°41′25″	Leaf, flower, soil
*Achillea*	19 Jul 2018		X	D- Goeppingen, meadow	2	354	7.8	48°40′57″	9°38′08″	Leaf, flower, soil

Type indicates plant compartment and soil sampled. Time series and spatial comparison highlight the samples used for the seasonal or spatial comparison. Samples of both plants in July 2016 were used for direct comparison and are marked with *.

**FIGURE 1:**
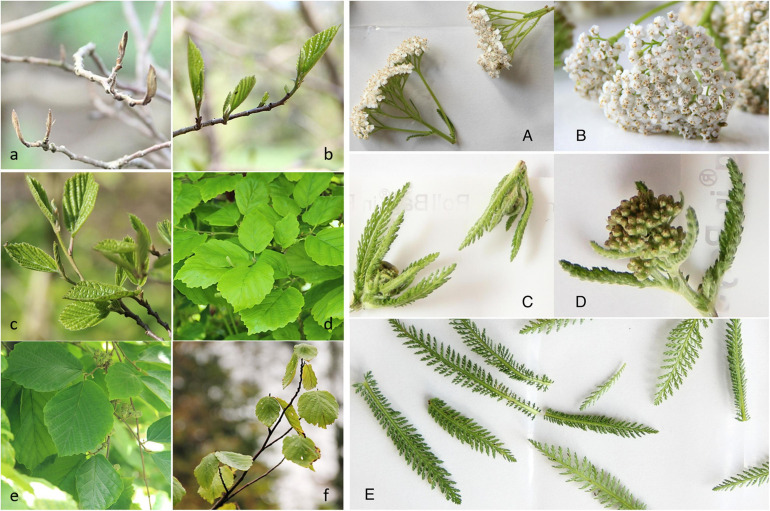
Plant organs of *Hamamelis virginiana* and *Achillea millefolium* studied in 2016. Left panel **(a–f)**: *Hamamelis*; **(a)**: Leaf buds (06 Apr); **(b)**: Leaf (18 Apr); **(c)**: Leaf (04 May); **(d)**: Leaf (20 May); **(e)**: Leaf (04 Jul); **(f)**: Leaf (20 Sep). Right panel **(A–E)**: *Achillea*: **(A,B)**: open flowers; **(C,D)**: Flower buds with upper young leaves; and **(E)**: leaves.

To ensure sufficient coverage of the phyllosphere, each biological sample contained leaves, flower buds or flowers of three individual plants growing in close proximity. Sampling was done in triplicate and at least 48 h after rainfall. Leaves of *Hamamelis* were collected at 1.2 to 2.0 m above ground. The herbaceous plant *Achillea* develops different leaves during the growing period. Thus, we first sampled leaves of the rosette in April and May and switched to the upper leaves of the stem in June and July (because rosette leaves decay until summer). The flower buds of *Achillea* in June were still closed and sampled together with their small surrounding young leaves (Figure 1). At each sampling also bulk soil was sampled in triplicate (top 20 to 70 mm) next to the plant below the canopy. Samples were stored at −80°C until analysis. The whole sampling procedure was carried out with gloves, DNA-free tweezers and DNA-free bags. Samples were transported in cooling bags at 4°C to the adjacent laboratory for further sample preparation.

### Differentiation Between Epi- and Endophytic Microorganisms

To obtain epiphytic communities all plant samples were run through a washing procedure by shaking (vortex) for 3 min in sterile 1x PBS-Silwet buffer; pH 7.4 (Silwet L77, final conc. 0.02%), followed by ultrasonication (640 W, Sonorex Digipuls, DL 510 H, Bandelin, Berlin, Germany) for 3 min. The buffer volumes were 100 ml for *Achillea* and 200 ml for *Hamamelis*, respectively. The sonication power was reduced to 80% (512 W) for young leaves from April to minimize cell damage. The washed-off epiphytic fraction from 2016 (time series) was centrifuged first in a 50 ml Falkon tube for 15 min at 4,369 × *g* followed by 12,900 × *g* in 2 ml tube (Eppendorf, Hamburg, Germany). In 2018 washed-off epiphytic fractions (spatial comparison) were filtered through a sterile 0.2 μm filter and stored at −80°C until DNA extraction. The endophytic plant samples, after ultrasonication treatment, were transferred in 50 ml Falkon tubes, rinsed additionally by shaking in fresh sterile PBS buffer to remove remaining epiphytes and stored at −80°C until extraction. For each extraction, PBS-Silwet buffer served as control.

### DNA-Extraction

To investigate the closely related and specialized endophytic community, all washed plant compartments were ground in liquid nitrogen just before DNA extraction. We used the DNeasy PowerSoil Kit (Qiagen, Hilden, Germany) following the manual to extract DNA. The final extracts were quantified using a Qubit 2.0 fluorometer (Invitrogen/ThermoFisher, Waltham, United States) and diluted with DNA-free water or concentrated by using Co-Precipitant Pink (Bioline, Memphis, TN, United States) following the manual if necessary. All samples were adjusted to a final concentration of 1–10 μg/μl DNA.

### Sequencing

Samples were sequenced by LGC Genomics GmbH (Berlin, Germany) on an Illumina MiSeq platform (Illumina, San Diego, United States). Fungi were amplified with primer ITS1F (5′-CTTGGTCATTTAGAGGAAGTAA-3′)/ITS2 (5′-GCTGCGTT CTTCATCGATGC-3′) targeting the ITS 1 region. Bacteria were amplified with primer pair 799f/1115r to reduce chloroplast-DNA signals. Controls were visually checked through an agarose gel.

### Bioinformatics

Datasets of the bacterial 16S rRNA gene and fungal ITS sequences were processed as follows: *Trimmomatic* 0.36 (Bolger et al., 2014) was used to truncate low quality read ends if the average quality dropped below 15. Primer sequences were removed from amplicon sequences using *bbduk*^1^. All datasets were subsequently processed with *USEARCH* v.10.0.240 (Edgar, 2010). Sequences were merged and low quality sequences were discarded [shorter than 300 base pairs (bp), accumulative sequencing error rate ≥1%] resulting in 9,652,175 and 9,128,731 high quality (HQ) amplicon sequences for the 799f–1115r primer set and the fungal ITS, respectively. HQ sequences were pooled according to the used primer set. In addition, sequences from the 16S rDNA dataset were truncated to equal length of 300 bp. Due to high variance in ITS sequence length this step was skipped for fungal ITS datasets. Subsequently, all sequences were dereplicated and sorted by abundance. Chimeric sequences were removed and remaining sequences clustered into zero-radius taxonomic units (ZOTUs) using the *unoise3* algorithm with a minimum unique sequence abundance of 8 across all samples. In total 20,133 (16S rDNA dataset) and 5,932 (ITS) ZOTUs were generated. All ZOTUs were taxonomically classified by alignment employing the *USEARCH* algorithm against the GTDB SSU database (Parks et al., 2018; release 89) for 16S rRNA gene sequences and the UNITE ITS database 8.0 (Abarenkov et al., 2020) with an *e*-value cut-off of 1e-10 and minimum sequence identity of 90% and *maxaccepts*/*maxrejects* option disabled. An abundance table was created by mapping HQ sequences of each sample to the ZOTUs.

### Statistical Analysis

Statistical analyses were performed using R v3.6.0 (R Core Team, 2018) with the packages *vegan* (Oksanen et al., 2017), *ape* (Paradis and Schliep, 2018), *drc* (Ritz et al., 2015), *ade4*, and *picante* (Kembel et al., 2010). Only samples with more than 1,000 mapped reads were considered for further analysis.

To account for varying sequencing depth, count tables of bacterial 16S rDNA and fungal ITS datasets were repeatedly rarefied to 1,000 sequences per sample (99 times). Subsequently, richness and Shannon entropy as well as species coverage were calculated for each iteration and the mean value was used for further analysis. Effective Number of taxa (EN) was calculated according to Jost (2006). Linear model fitting was used to determine a relationship between richness and EN of the associated leaf community to the growth period of *Achillea* and *Hamamelis. P*-values < 0.05 were considered significant. Differences between richness and diversity of the microbiome of different plant compartments were tested using analysis of variance (ANOVA) and Tukey′s honest significant difference test after checking for normal distribution (Shapiro–Wilk test) and homoscedasticity (Fligner test).

Prior to further analysis unrarefied samples were converted to relative abundances by dividing individual ZOTU counts by the total number of reads per sample. To determine core and specific plant species/tissue community, only bacterial and fungal taxa that occurred in at least two triplicates or in more than 50% of the replicates, if more than three replicates were used, were considered. Bray–Curtis distances (Bray and Curtis, 1957) of bacterial and fungi abundances at each location were visualized by non-metric multidimensional scaling (NMDS; *k* = 2; 999 permutations). A permutational multivariate analysis of variance (PERMANOVA) with 9,999 permutations was performed to determine the dataset variance explained by sample type, plant species, tissue, and soil pH. The core microbiome of the four locations was determined only for the July samples of *Achillea*. To exclude rare taxa in the core microbiome analysis, all bacterial and fungal genera, which occurred with at least 0.01% of the total community in more than two thirds of a particular sample type (leaf epi, leaf endo, flower epi, or flower endo) at one location, were considered to be present. Mantel tests (9,999 permutations) using Bray–Curtis distances were used to determine correlation between bacterial and fungal datasets.

### Soil Analysis

Wavelength-Dispersive X-Ray Fluorescence (XRF; Axios FAST, Malvern Panalytical, Malvern, United Kingdom) analyses was used to determine the concentrations of selected major (Si, Al, Fe, Mg, Ca, N, K, P) and trace (Mn, As, Co, Cr, Cu, Mo, V, U, Zn, Zr) elements of soil samples. For each analysis 0.7 g of dried and sieved (2 mm) sample were used and measured according to Atar et al. (2019). Major and trace elements are reported as weight% and ppm, respectively.

Soil pH was measured in an aqueous suspension (soil:deionized water, 1:2.5, v:v) using a InLab Micro (Mettler Toledo, Giessen, Germany).

## Results

**FIGURE 2:**
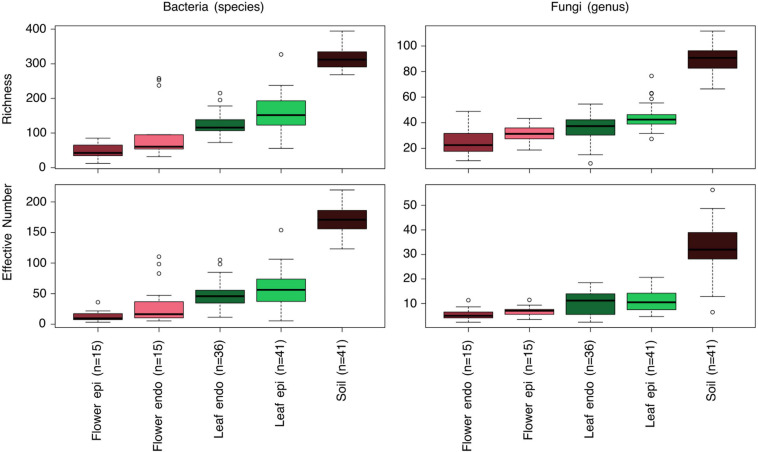
Box-Whisker plots of Richness and Effective Number diversity of bacterial and fungal epi- and endophytic communities of the flower and leaf compartments as well as soil of *Achillea* and leaf and soil of *Hamamelis*. Given are mean values, quantiles 2 and 3 (box) and 1 and 4 (lines) and outliers (<5% and >95% of the mean). In parenthesis: number of samples. Samples were subsampled (repeatedly rarefied to 1,000 sequences) for comparison. For statistical comparisons of these data see Supplementary Table 1.

We assessed the microbial community of 45 soil and 120 plant samples from above ground, comprising epiphytic and endophytic communities of *Achillea* (24 leaves, 3 flower buds, 15 flowers) and *Hamamelis* (18 leaves). The soil communities showed the highest richness and EN diversity of bacterial species and fungal genera compared to plant samples, the latter exhibiting a decreasing diversity from epiphytic leaf to endophytic flower communities. Richness of each of the epiphytic communities was significantly higher than that of their corresponding endophytic compartment, except for fungi on flowers (ANOVA and *post hoc* test, *p* < 0.05). The EN differed only significantly between epi- and endophytic bacterial communities on leaves (*p* < 0.05; Figure 2; Supplementary Table 1). As shown by rarefaction analysis, sequencing efforts of most of the plant samples and also of the highly diverse soil samples reached almost saturation (Supplementary Figure 1).

### The Microbiome of *Achillea* and *Hamamelis*

**FIGURE 3:**
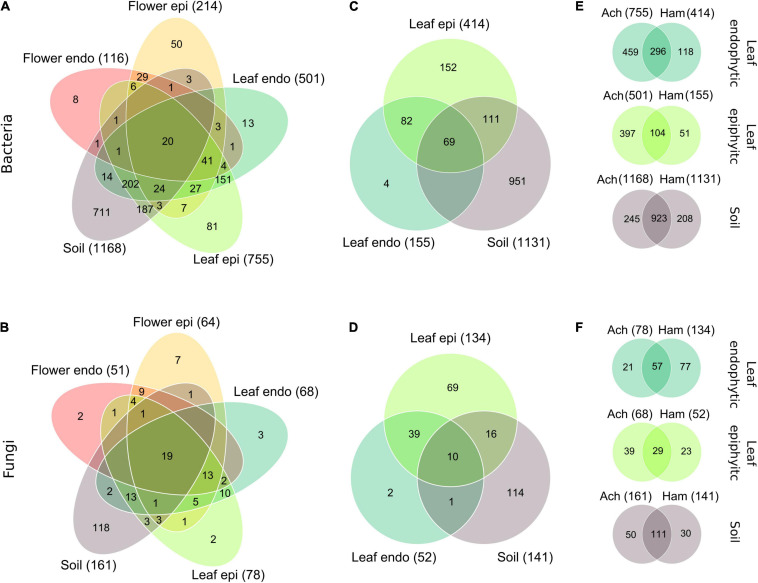
Venn-diagrams of the partitioning of the bacterial species and fungal genera of *Achillea* **(A,B)** among the epi- and endophytic leaf, flower and the soil compartments, and of *Hamamelis* **(C,D)** among the epi- and endophytic leaf and the soil compartments in July in the WALA garden in 2016 (location 1a). Partitioning of the bacterial species **(E)** and fungal genera **(F)** of the endo- and epiphytic leaf and soil communities between *Achillea* (Ach) and *Hamamelis* (Ham) is also shown. Intersection without number: detected taxa = 0. In parenthesis: number of compartment-specific taxa.

In July, *Achillea* starts to bloom and *Hamamelis* has fully developed leaves. Therefore, we assessed the microbiome of both plants in the WALA garden (location 1a) at this time. *Hamamelis* blooms in winter, consequently, flowers of this plant were not included in this survey. As described for the overall assessment (see above) both plants at this location and time also showed a decreasing number of total detected bacterial and fungal taxa from soil to epi- and endophytic leaf and further for *Achillea* to the epi- and endophytic floral communities (Figures 3A–D).

Besides this generally decreasing number of total bacterial and fungal taxa, proportions of compartment-overlapping taxa of *Achillea* decreased from soil to leaf and flower as indicated by the numbers in intersecting areas of Figures 3A,B. Forty-eight percent of all 878 plant-associated bacterial species were exclusive to plant tissue (28% to leaf, 10% to flower, and 10% to both) and not detected in soil. Respective proportions of fungi were even higher as 58% of all 102 plant-associated fungal genera were exclusive to plant tissue (15% to leaf, 18% to flower, and 25% to both). Only 7% of all bacterial species and 31% of all fungal genera associated with plant tissue were shared among all plant compartments (Figures 3A,B). For *Hamamelis*, 57% of all 418 leaf-associated bacterial species and 80% of all 137 leaf-associated fungal genera were exclusive to the plant compartments and not detected in soil. Thus, *Hamamelis* harbored a higher relative proportion of leaf-exclusive bacteria and in particular of fungi than *Achillea* (Figures 3C,D).

*Hamamelis* and *Achillea* grew in close proximity (max. 10 m distance) in the WALA garden. The composition of the soil communities of both plants showed high similarity, the bacterial communities by more than 79% and the fungal communities by more than 68% (Figures 3E,F). Pronounced differences, however, were detected in the leaf microbiome of both plants. Leaves of *Achillea* exhibited a much higher total number of bacterial species and endophytic fungal genera than *Hamamelis*, whereas the total number of epiphytic fungal genera was higher in leaves of the latter (Figures 3E,F).

### Seasonal Leaf Diversity

**FIGURE 4:**
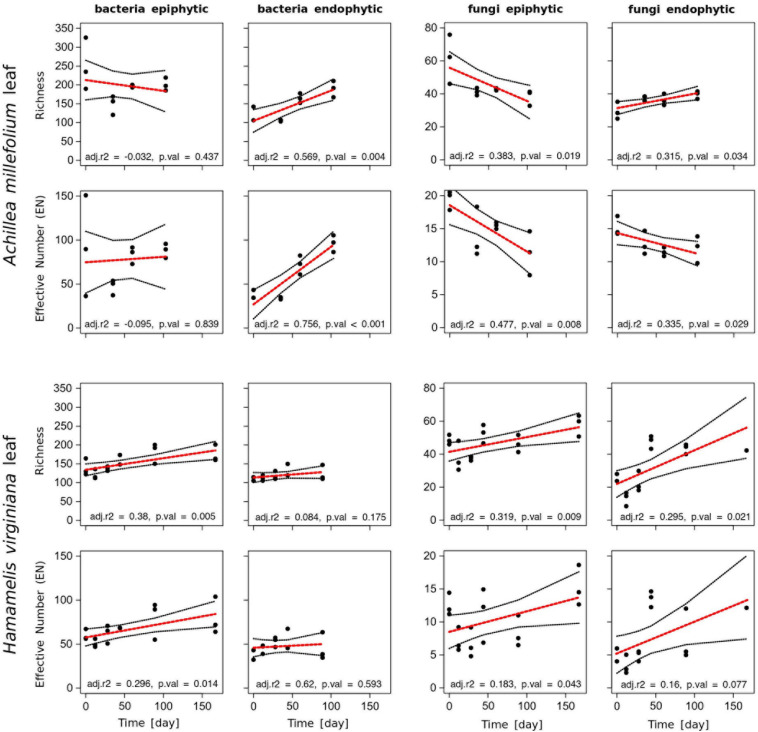
Richness and Effective Number EN of epi- and endophytic bacterial (species) and fungal (genera) communities of leaves of *Achillea* and *Hamamelis* between April (*t* = 0) and September 2016 (*t* = 180) in the WALA garden (location 1a). The dataset was subsampled to 1,000 sequences per sample. The red line fits a linear model with adjusted *r*^2^ value (adj. *r*^2^) and significance (*p*. val). Missing values for *Hamamelis* in September (day 167) did not pass the threshold of 1,000 reads and were therefore excluded from this analysis.

To examine whether leaf development over the season also affected the associated microbial communities, we analyzed time series of the microbial leaf communities of *Achillea* and *Hamamelis* collected in the WALA garden. At the first sampling point in April, the endophytic bacterial and fungal communities exhibited a lower richness and EN than the epiphytic communities (Figure 4). The epiphytic bacterial community of *Achillea* revealed some temporal fluctuations but no consistent temporal pattern. However, the richness and EN of the endophytic bacterial community increased consistently over time (Figure 4). In contrast, the fungal leaf community of *Achillea* decreased over time except in richness of the endophytic community (Figure 4). At the last sampling point in July, richness and EN of the epi- and endophytic communities of bacterial species and fungal genera were similar (Figure 4).

For *Hamamelis*, different patterns were observed. Richness and EN of the endophytic bacterial leaf community remained similar over time, whereas both variables of the epiphytic community increased significantly, finally yielding higher values than the endophytic community (Figure 4). The fungal epi- and endophytic leaf communities of *Hamamelis* increased over time as well, but temporal fluctuations were rather high (Figure 4).

**FIGURE 5:**
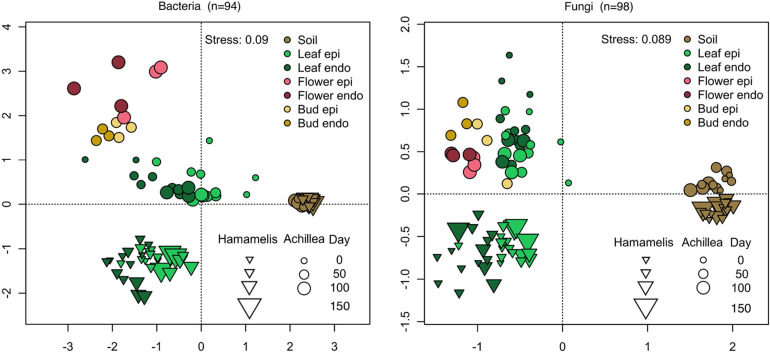
NMDS analysis of bacterial (left) and fungal (right) communities of the time series of *Achillea* (April to July) and *Hamamelis* (April to September). Shown are communities of soil, endophytic (endo) and epiphytic (epi) leaf and flower communities in the WALA garden 2016 (location 1a). *n*: number of samples.

An NMDS analysis confirmed the temporal patterns and changes of both microbial communities of both MPs (Figure 5). This analysis was extended to the microbial communities of the soils of both MPs and to the flower buds and flowers of *Achillea*. It revealed distinct and temporally stable soil communities of both MPs. The epi- and endophytic leaf communities of *Hamamelis* were slightly different with some temporal variations. For *Achillea* the NMDS analysis revealed distinct leaf-, bud- and flower-associated bacterial communities. The epiphytic fungal community was slightly but consistently different from the respective endophytic community. A PERMANOVA considering only plant-associated samples revealed the plant species as the major determinant explaining the differences in the bacterial and fungal communities, 34% for bacteria and 40% for fungi. The tissue type (leaf or flower) covered 23% and 17% of the variation of bacteria and fungi, respectively. The distinction in epi- and endophytic communities was not significant and explained less than 3% of the community variance (Supplementary Figure 2).

### Community Composition and Seasonal Dynamics

**FIGURE 6:**
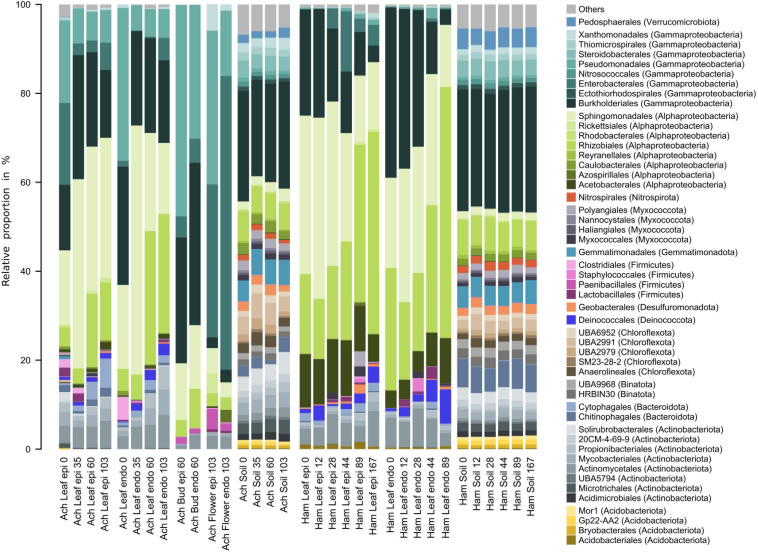
Relative proportions of the 50 most abundant bacterial orders of the epi- and endophytic leaf, bud, flower and soil communities of *Achillea* (Ach) over a period of 103 days (April to July) and of the epi- and endophytic leaf and soil communities of *Hamamelis* (Ham) over periods of 89 and 167 days (April to July and September), respectively.

**FIGURE 7:**
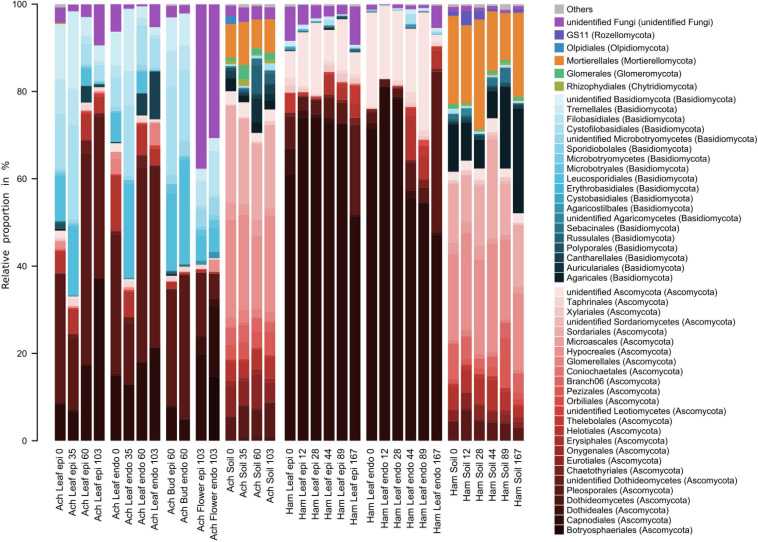
Relative proportions of the 50 most abundant fungal orders of the epi- and endophytic leaf, bud, flower, and soil communities of *Achillea* (Ach) over a period of 103 days (April to July) and of the epi- and endophytic leaf and soil communities of *Hamamelis* (Ham) over periods of 89 and 167 days (April to July and September), respectively.

Characteristics and temporal dynamics of the microbial communities of *Achillea*, *Hamamelis* and the associated soil between April and September were clearly reflected in the 50 most abundant bacterial and fungal taxa (Figures 6, 7 and Supplementary Figures 8, 9). As indicated above for the entire communities also the most abundant taxa exhibited a much greater diversity of the soil-associated microbial communities than the above-ground plant-associated communities.

Bacterial communities of both MPs were composed of *Alphaproteobacteria*, *Gammaproteobacteria*, *Actinobacteria*, *Firmicutes*, *Deinococcota*, and *Bacteriodota* (Figure 6). *Alphaproteobacteria* were most abundant with 40 to over 60% of total abundance on leaves and higher proportions on *Hamamelis* than on *Achillea*. *Sphingomonadales* and *Rhizobiales* constituted major alphaproteobacterial fractions and increased in abundance over the progressing season. Furthermore, *Acetobacterales* were detected on leaves of *Hamamelis* and constituted up to 12% of total abundance. *Gammaproteobacteria* were the second most abundant group on leaves of both MPs and on *Achillea* even dominated initially on leaves, flower buds and flowers to more than 70%. On leaves of *Hamamelis*, *Gammaproteobacteria* decreased in proportion and finally constituted less than 15% in the epiphytic and below 5% in the endophytic communities. *Burkholderiales* were the most abundant order of this subclass on leaves of both plants and on flower buds of *Achillea*. *Pseudomonadales* and *Enterobacterales* also constituted substantial proportions, in particular in spring on leaves and the latter on flowers constituting 35% of the epiphytic and 70% of the endophytic community. Further, *Actinobacteria* with several orders, *Firmicutes*, mainly their orders *Clostridiales*, *Lactobaccilales* and *Phaenibacillales*, *Bacteriodota*, mainly their order *Cytophagales*, and *Deinococcota* with their order *Deinococcales* were consistently present on leaves of both MPs. *Actinobacteria* constituted between 5 and 18% and the other groups not more than 7% each (Figure 6).

Genera such as *Sphingomonas, Methylobacterium*, *Massilia, Herbaspirillium*, *Kineococcus*, and *Deinococcus* were shared by both MPs but leaves of each plant exhibited specific patterns, which differed in relative proportions of these genera (Supplementary Figure 8). Plant-specific seasonal dynamics were also recorded. On *Achillea* leaves, relative proportions of *Methylobacterium*, *Kinneococcus* and *Spirosoma* increased over time and those of *Pseudomonas*, *Janthinobacterium* and FW-11 decreased. Few genera like *Gilliamella*, *Conexibacter*, and *Microvirga* exhibited higher proportions in the epiphytic relative to the endophytic leaf community of *Achillea* in April and compared to later time points (Supplementary Figure 8). On *Hamamelis* leaves, *Deinococcus* and *Microvirga* increased their relative proportions over time and those of *Herbaspirillium, Burkholderia* and *Massilia* decreased. *Gilliamella* exhibited a higher proportion of the epiphytic leaf community of *Hamamelis* relative to *Achillea* (Supplementary Figure 8).

The dominant bacteria of the associated soil of both plants were almost identical and showed only minor temporal variations (Figure 6). Some typical plant taxa were not detected or were exceedingly rare in soil such as *Methylobacterium*, *Kinneococcus, Lactobacillus*, and *Deinococcus* (Figure 6 and Supplementary Figure 8).

Fungal communities of both MPs and the associated soils were clearly different (Figure 7 and Supplementary Figure 9). On leaves of *Hamamelis*, taxa of the phylum *Ascomycota* dominated by over 90% of total fungal abundance, in particular the order *Capnodiales*. On *Achillea*, taxa of the phylum *Basidiomycota* and various orders were also prominent and constituted proportions of roughly 20 to about 60% (Figure 7). Highest proportions of taxa of this phylum were detected on leaves at the second sampling date (May) when new rosette leaves had developed, and the first stem growth appeared as well as later on flower buds. Respective proportions of *Ascomycota* on these dates and compartments were below 40%. On flowers, unidentified fungi constituted ∼35% of total abundance whereas they constituted <10% on buds and leaves (Figure 7). Fungi also showed a clear plant-specific community, but several dominant genera also overlapped between both plants such as *Didymella, Mycosphaerella* and *Cladosporium* but often with a plant-specific dominance (Supplementary Figure 9). The fungal communities of *Hamamelis* were dominated by the genus *Microcyclospora*, an unidentified Capnodiales, *Uwebraunia* and *Xenosonderhenia*, whereas *Achillea* harbored other dominant fungi, such as *Leucosporidium, Septoria, Filobasidium, Tetracladium*, and *Stragonosporopsis*.

The fungal leaf communities of *Achillea* underwent seasonal variations. Basidiomycotal genera such as *Leucosporidium*, *Filobasidium* and *Dioszegia* relatively decreased in both epi- and endophytic leaf communities during the growing season (Supplementary Figure 9). In contrast, ascomycotal genera such as *Didymella, Boeremia* and *Ramularia* increased, together with the basidiomycotal genus *Thanatephorus* (Supplementary Figure 9). Only the latter was enriched in the endophytic leaf community at the final sampling point in July, together with an unidentified member of the *Plectosphaerellaceae*. The composition of the fungal leaf communities of *Hamamelis* remained rather stable seasonally. However, *Capnodiales* relatively decreased during summer in the endophytic leaf community and exhibited reduced numbers in the epiphytic as well at the last sampling date in September. At this date, *Dothideomycetes* with its lineages *Didymella*, *Mycosphaerella* and *Epicoccum* increased concomitantly (Supplementary Figure 9).

Fungi detected in soil were mostly absent or present in low abundances in the above-ground plant compartments. The fungal soil communities were dominated by 50 to 80% of total abundance by various orders of *Ascomycota* (Figure 7). *Basidiomycota* constituted <30% and in most cases <15%. Further, *Mortierellales*, not at all detected in the MPs above ground, constituted ∼8 to 23% in the soil of both plants with higher proportions in that of *Hamamelis*. Other and unidentified fungi of generally minor proportions revealed some differences between the soils of both MPs (Figure 7). A few dominant genera were only detected in the soil of *Achillea*, such as *Didymella* and *Tetracladium*, whereas three genera of the *Clavariaceae* (*Clavaria*, *Clavulinopsis*, one unidentified) dominated in *Hamamelis* soil (Figure 7 and Supplementary Figure 9).

On *Achillea*, the number of genera among the most abundant 50 bacterial and fungal taxa decreased from leaves to flower buds and flowers (Supplementary Figure 8). Two enterobacterial genera, *Nissabacter* and *Pectobacterium*, and one fungal genus, *Botryosphaeria*, were detected exclusively on flowers and even as prominent community members (Supplementary Figures 8, 9).

To explore the connection between fungal and bacterial community we computed Mantel correlations of Bray–Curtis distances between both communities for all soil, *Achillea* and *Hamamelis* samples. Communities present in *Achillea* (Mantel’s *r* = 0.59, *p* < 0.001) and soil (Mantel’s *r* = 0.49, *p* < 0.001) were tightly linked whereas communities from *Hamamelis* (Mantel’s *r* = 0.21, *p* = 0.002) showed a weaker but still significant correlation.

### Effect of Location on Composition of Microbial Communities of *Achillea*

**FIGURE 8:**
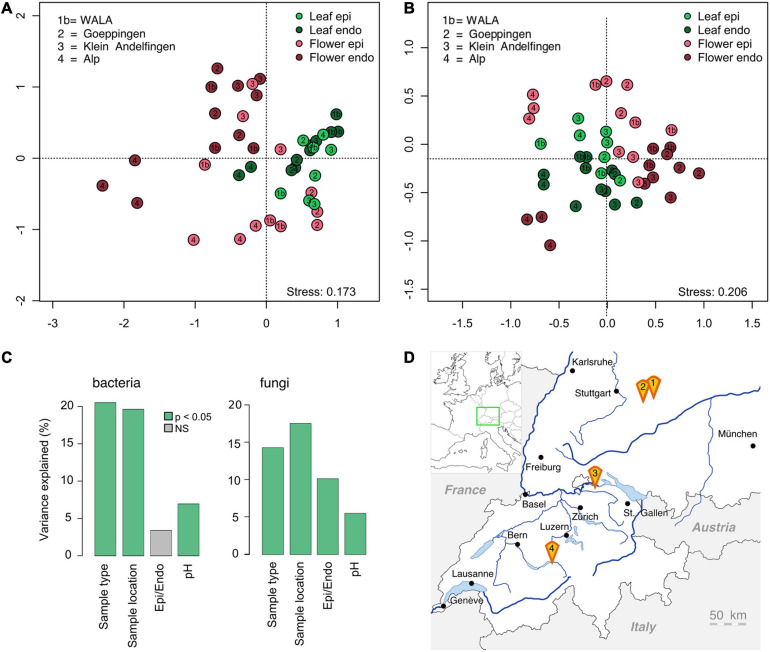
Graph of the NMDS analysis of the bacterial [**(A)**; *n* = 43] and fungal epi- and endophytic leaf and flower communities [**(B)**, *n* = 44] of *Achillea* at four different locations in southern Germany and Switzerland **(D)**. For details of locations see Table 1. **(C)** PERMANOVA (*p* < 0.05) including sample type (leaf/flower), location, epi- and endophytic compartment and soil pH of each location.

To investigate the geographic impact on the microbial communities of *Achillea*, soil, leaves and flowers were collected in July 2018 at four locations in southern Germany and Switzerland, up to 250 km apart. The sites differed by altitude, soil pH (Table 1 and Figure 8D) and inorganic soil elements (Supplementary Figure 3). Locations 2 and 3 exhibited pronounced differences in their relative proportions of Ca and Si as compared to locations 1b and 4, which are biodynamically cultivated. A PERMANOVA revealed that location explains 18% of the variance of bacterial and fungal plant communities, closely followed by plant compartment (leaf or flower), explaining 18% and 14% of the variance of bacterial and fungal communities, respectively. Differences in the epiphytic and endophytic sub-communities were only significant for the fungal communities and explained 10% of the variance (Figure 8C). Soil pH explained only 5% of the plant-community variance.

The composition of the soil-associated microbial communities was location-specific (Supplementary Figure 4). These patterns of the microbial soil communities were not reflected in the respective plant communities as illustrated by an NMDS-analysis (Figures 8A,B). Clustering of the leaf- and flower-associated microbial communities was mostly compartment-specific and only the fungal and endophytic bacterial floral communities at location 4 were distinctly different from those of the other locations (Figure 8 and Supplementary Figure 7).

### Core Community and Unique Taxa of Plant Compartments of *Achillea* and Soil

To elucidate general and location-specific aspects of the microbiome of *Achillea*, we assessed the microbial core community and unique taxa of a given location. We defined the core community as a subset of taxa occurring at all locations and unique taxa as those found only at one location and more specific in a plant compartment or soil. The analysis is based on all detected taxa that occurred in at least two of the triplicates. Numbers of core taxa decreased from soil to flower and from epiphytic to endophytic compartments (Table 2) and reflect the general patterns described above. Numbers of total and unique taxa followed the same general pattern, except for the epiphytic bacterial floral communities which exhibited the highest number of taxa (Table 2) possibly reflecting that the flower is affected greatest by environmental impacts and possible transmission by microbes including pollinating insects. The proportion of bacterial core taxa as percent of total bacterial abundance decreased less from soil to flower than their percentage as number of total bacterial taxa. Respective percentages of fungi even increased (Table 2). The fraction of bacterial core taxa as percent of total abundance in the plant compartments ranged between 54 and 95% not exceeding 29% as fraction of the total number of bacterial taxa. Percentages in the endophytic compartments were consistently higher than in the epiphytic compartments. In the endophytic bacterial floral community with the lowest number of taxa, the 28 core genera comprised only 17% of the number of total bacterial taxa but almost 76% of total bacterial abundance. Accordingly, the 19 core genera of the fungal endophytic floral community constituted 18% of the number of total genera but 95% of the total abundance (Table 2).

**TABLE 2:** Detected bacterial species and genera and fungal genera of the different plant compartments of *Achillea* at four different locations (for locations see Table 1).

Compartment	Total taxa	Sum of unique taxa of one location	Unique taxa at one location%	Core taxa, absolute	Core taxa,% of total taxa	Core taxa,% of total abundance
**Bacteria species**						
Soil	1985	465	0.7	836	42.1	93.7
Leaf epiphytic	913	334	2.2	230	25.2	80.5
Leaf endophytic	659	307	2.4	113	17.1	90.1
Flower epiphytic	1002	612	9.0	80	8.0	54.2
Flower endophytic	329	163	1.9	34	10.3	64.9
**Bacteria genera**						
Soil	826	150	0.2	438	53.0	97.2
Leaf epiphytic	409	133	0.3	120	29.3	90.1
Leaf endophytic	263	103	1.9	59	22.4	94.6
Flower epiphytic	451	236	1.8	73	16.2	73.7
Flower endophytic	170	77	0.8	28	16.5	76.1
**Fungi genera**						
Soil	292	111	4.4	64	21.9	65.4
Leaf epiphytic	160	52	1.8	40	25.0	91.5
Leaf endophytic	163	64	5.4	42	25.8	88.5
Flower epiphytic	213	112	1,0	30	14.1	86.5
Flower endophytic	104	56	1,6	19	18.3	94.6

*Shown are numbers of total taxa, unique taxa (occurring at only one location) and their relative abundance (% of the total community), core taxa and their percentages of the total number of taxa and the total abundance of the community. A taxon was considered as core when it was detected in at least 66% of the replicates.*

As the pure “core” number does not reflect whether a core taxon is specific to one compartment or included in the core community of another one, we also assessed the compartment-specific core taxa, comprising all taxa solely detected in a specific compartment. Soil samples revealed the largest compartment-specific core community, both in total number and in relative abundance (Supplementary Table 2). Compartment-specific bacterial epiphytic core communities revealed more taxa than endophytic core communities, and no bacterial genus was exclusively detected in the endophytic core community of leaves. Five genera were exclusive to the endophytic floral community of which three belonged to *Enterobacteriaceae* (Supplementary Tables 2, 3). Regarding fungi, nine and five genera occurred exclusively in the epiphytic and endophytic core community of leaves, respectively. Only three basidiomycotal genera occurred exclusively as core genera of the epiphytic flower community (Supplementary Tables 2, 4).

An even more rigorous analysis excluding any detection in soil revealed a plant-exclusive endophytic core community in leaf and flower of two and three bacterial genera and eight and six bacterial species, respectively, (Supplementary Table 2, plant core never in soil). Similarly, four and one of the endophytic fungal core genera were never detected in soil (Supplementary Table 2, plant core never in soil). These results show that the core microbiome of *Achillea* harbors soil-independent plant compartment-specific core taxa in both its endophytic and epiphytic compartments.

## Discussion

Since the advent of high throughput sequencing our knowledge and understanding of the composition and diversity of bacterial and fungal communities of the various plant compartments above ground, the rhizosphere and the associated soil has greatly improved. The general pattern emerged that the diversity decreases from below ground to stem, twig, leaf and flower with the strongest decrease from below to above ground (Ottesen et al., 2013; Gomes et al., 2018; Hamonts et al., 2018; Wei and Ashman, 2018; Grady et al., 2019; Comeau et al., 2020; Massoni et al., 2020), even though different trends for leaf and flower were also reported, e.g., for the endemic Hawaiian tree *Metrosideros polymorpha* (Junker and Keller, 2015) and wild strawberries (Wei and Ashman, 2018). In quite a few studies only one or two plant compartments and soil were investigated and even fewer studies investigated prokaryotes and fungi simultaneously (e.g., Hamonts et al., 2018; Comeau et al., 2020). A distinction between epi- and endophytic communities was made even more rarely (e.g., Gomes et al., 2018; Yao et al., 2019) and not yet for prokaryotes and fungi of the same plant. Further, there is still limited information available on the temporal development of plant-associated microbial communities. For a better understanding of the colonization patterns and how they evolve and establish it is important to study these features from below ground to leaf and flower at one plant species.

Therefore, we investigated the epiphytic and endophytic bacterial and fungal communities of leaves, flower buds and flowers of *Achillea* and the respective epiphytic and endophytic microbial communities of leaves of *Hamamelis* including the associated soil communities. Flower buds and flowers of *Hamamelis* were not considered because this deciduous shrub flowers in winter. Overall richness and diversity of bacterial and fungal taxa decreased from soil to leaves of both MPs and further to flowers of *Achillea*, ending in a specialized flower-associated microbial community of a few dominant members reflecting presumably the shorter life time of flowers as compared to leaves. Such a reduction in richness from leaf to flower has also been reported for bacterial communities in a very recent study on several wild flowers (Massoni et al., 2020) and of tomatoes which further showed that richness of top leaves was similar to that of flowers (Ottesen et al., 2013). In wild strawberries, however, significant differences of the richness of bacterial communities between leaves and flowers were not found (Wei and Ashman, 2018). Similarly, fungal communities associated with leaf and flower of *C. sativa* neither exhibited differences (Comeau et al., 2020). Studying the endemic Hawaiian tree *M. polymorpha* Junker and Keller (2015) found a higher bacterial diversity on flower organs than on leaves but a higher Shannon index on the latter. These findings indicate that despite the general decrease in diversity from below to above ground different plants exhibit distinct trends, obviously reflecting intrinsic features such as their specific habitus, e.g., tree, shrub, herbaceous, perennial or annual plant, size, and developmental time. Environmental and climatic factors may further contribute to shaping the plant microbiome.

### Seasonal Patterns of Microbial Leaf Communities

The seasonal patterns of the epi- and endophytic leaf communities of bacteria and fungi of both MPs exhibited pronounced differences, obviously reflecting the distinct habitus and host-microbe interactions of each MP. In spring, *Achillea* first develops basal rosette leaves in close contact to the soil; further leaves follow along the growing stem until flower buds are produced. All leaves are relatively short-lived and in contrast to the arborescent shrub *Hamamelis* do not bud out of the twigs well above the soil. Leaves of *Hamamelis* are generally bigger, sturdier and persist throughout the season from April to November. Richness and EN of *Achillea*’s epiphytic bacterial community was highest on the rosette leaves at the first sampling and dropped strongly at the second sampling. Presumably, most of these epiphytic bacteria were transferred from soil, which can be considered as key reservoir or seedbank of leaf microbes (Copeland et al., 2015; Grady et al., 2019). Our NMDS analysis supports this assumption, as the composition of the early epiphytic bacterial community was most similar to that of soil (Figure 5). The strong decline in richness and diversity of this bacterial community at the second sampling of young leaves on the stem seems to reflect that these leaves develop newly without direct contact to the soil and rather little environmental exposition. The slight increase in richness thereafter may reflect that these leaves accumulated bacteria transmitted from the environment. The endophytic bacterial leaf community of *Achillea* continuously increased from April to July, indicating that the endophytic leaf compartment provided niches for the successful establishment of new taxa in the progressing season. In contrast to bacteria, endo- and epiphytic fungal leaf communities of *Achillea* decreased in EN diversity and the epiphytic fungal community also in richness from April to July, indicating that a substantial fraction of the fungi associated with the young leaves early in the season did not establish a long term relationship with the MP. As discussed for epiphytic bacteria most of the fungi were presumably transferred from soil to the leaf-rosette, persisted in early leaf stages but did not establish.

For *Hamamelis* the seasonal development of the bacterial and fungal leaf community was quite different. Whereas the richness and EN diversity of the epiphytic bacterial leaf community increased slightly from April to July, these indices remained almost constant for the endophytic communities. As also the temporal colonization patterns of the bacterial community changed only slightly, these findings indicate that only few new bacteria were able to settle and dwell on leaves after their initial development after budding (Figures 4–6). It appears that bacterial leaf communities of *Hamamelis* reached a stable composition shortly after early leaf development possibly promoted by certain bacterial taxa transferred from adjacent bark and branch communities. It has been reported from other woody plants and trees that bacterial bark and branch communities are more diverse than the leaf communities (Leff et al., 2015; Harrison and Griffin, 2020). Richness and EN of the fungal leaf communities increased from April to July and that of the epiphytic community further until September, reflected also in the taxonomic colonization pattern (Figures 4, 5, 7). Settling and establishing of new bacterial taxa was of minor significance in *Hamamelis*, whereas the fungal leaf community responded positively to the leaf development over time by increasing its diversity.

Reasons for these differences in the temporal colonization patterns of the bacterial and fungal leaf communities of *Achillea* and *Hamamelis* presumably include differences between the size, development and physiology of a herbaceous perennial plant and a deciduous arborescent shrub. Both MPs provide their respective microbiome with distinct substrates and/or growth-promoting and growth-inhibiting compounds which may also lead to distinct interactions of their bacterial and fungal components (Da Silva et al., 2000; Candan et al., 2003; Saeidnia et al., 2011; Saénchez-Tena et al., 2012; Duckstein et al., 2012a; Apel et al., 2021). The Mantel test yielding closer interactions of the bacterial and fungal communities on *Achillea* than on *Hamamelis* reflects these features. Various and in particular plant-specific modes of interactions among the residing microbial communities appear to contribute to these differences (Hardoim et al., 2015; Jia et al., 2016; Brader et al., 2017). Various leaf-associated fungi have been shown to produce antibiotic compounds inhibiting bacterial growth in the phyllosphere (Taghinasab and Jabaji, 2020), possibly contributing to the predominance of fungi on the leaves of *Hamamelis*. During leaf development of *Hamamelis* the proportions of condensed and hydrolysable tannins increase (Rehill and Schultz, 2012) and the pH of the leaf cytoplasm is rather low (pH ∼4.0, S. Sauer, unpubl. data). This may favor the growth and diversification of fungal communities, suggesting that *Hamamelis* selects for its specific microbial leaf community. Presumably, weather conditions had little impact as both plants grew closely together.

These findings broaden our knowledge on the temporal development of microbial phyllosphere communities as only few studies addressed this important issue and none an MP or bacterial and fungal communities simultaneously. A study, which examined the development of bacterial leaf communities of bean, canola and soybean over 54 days, reported a decreasing diversity over this period (Copeland et al., 2015). However, these authors did not distinguish between epi- and endophytic compartments and identified rainfall as the most important variable affecting temporal changes. A varying richness over the growing season from April to October with most pronounced diversity changes early in the season was observed for leaf-associated prokaryotic communities of switchgrass and miscanthus, important biofuel crops in North America (Grady et al., 2019). Pronounced differences between spring and fall were reported for the diversity and composition of epi- and endophytic fungal communities of leaves and twigs of olive trees in the Mediterranean region with a generally higher diversity in spring as compared to fall and of the epiphytic community relative to the endophytic community (Gomes et al., 2018). As spring is the main growing season in the Mediterranean these results are in line with ours of *Hamamelis* showing that diversity of leaf-associated fungal communities increases in the growing season even though our results show a higher temporal resolution. Interactions of bacteria and fungi regarding promotion and inhibition of growth of members of either community and the host are very important in plant-associated microbial communities (Hardoim et al., 2015; Müller et al., 2016; Brader et al., 2017; Caruso et al., 2020). Therefore, it is important to obtain more information on the simultaneous colonization patterns of both microbial communities to better elucidate the significance of their interactions for plant health and in MPs.

### Microbial Taxa of the Plant and Soil Communities

Our results show that each MP harbors a specific bacterial and fungal microbiome. For *Achillea* we further showed that the leaf-, bud- and flower-associated microbial communities were composed of a different array of taxa, even though some were also present in adjacent compartments. Other comparative studies have shown that the microbiome of different plants is distinct, reflecting the specific feature of the given plant and its environment (Copeland et al., 2015; Lumactud and Fulthorpe, 2018; Massoni et al., 2020). The various microbial taxa include commensals, potential pathogens but also probiotic and host growth-promoting members interacting with other microbes and the host plant in various mutualistic and antagonistic and still little understood ways (Hardoim et al., 2015; Berg et al., 2016; Brader et al., 2017; Castronovo et al., 2020).

The bacterial taxa we detected in soil and the plant compartments of both MPs are rather typical as similar phylogenetic groups have also been detected on other plants. Members of *Sphingomonadales* and *Rhizobiales* of *Alphaproteobacteria* are typical leaf-associated bacterial taxa (Copeland et al., 2015; Hardoim et al., 2015; Hamonts et al., 2018; Pontonio et al., 2018; Wei and Ashman, 2018; Grady et al., 2019; Comeau et al., 2020; Massoni et al., 2020). Additionally, *Actinobacteria* are typical leaf-associated prokaryotes but often constitute high proportions in the rhizosphere and in soil (Copeland et al., 2015; Hardoim et al., 2015; Pontonio et al., 2018; Wei and Ashman, 2018; Vokou et al., 2019; Comeau et al., 2020; Massoni et al., 2020). A continuous temporal relative increase of *Actinobacteria* in the endophytic leaf community, as we observed for *Achillea*, has already been reported for bean, soybean and canola (Copeland et al., 2015). Endophytic *Actinobacteria* may be of particular importance in MPs as they produce a diverse range of bioactive compounds (Golinska et al., 2015). *Gammaproteobacteria* and in particular their orders *Pseudomonadales* and *Burkholderales* are further prominent members of the leaf and soil communities and may exhibit plant growth promoting features (Hardoim et al., 2015; Castronovo et al., 2020; Comeau et al., 2020; Massoni et al., 2020). They were abundant members of the leaf communities of both MPs, but differences on the genus level were monitored, indicating that each plant selects for its specific bacterial community. As our and data from other studies show, bacteria can also be prominent members of the flower communities, in particular *Enterobacteriaceae* (Shade et al., 2013; Aleklett et al., 2014; Junker and Keller, 2015; Wei and Ashman, 2018; Castronovo et al., 2020). In fact, *Enterobacteriaceae* appear to be specifically associated to the style and stamen of the flower as reported for the tree *M. polymorpha* (Junker and Keller, 2015). Interestingly, the nitrogen-fixing *Acetobacter* and further putative N_2_-fixing *Rhizobiales* such as *Devosia*, *Bosea*, and *Roseomonas* were prominent on leaves of *Hamamelis* and very rare on leaves of *Achillea*, suggesting that the Nitrogen demand, supply and metabolism of *Hamamelis* was different from that of *Achillea*. The leaves of *Hamamelis* presumably provide a favorable environment for these bacteria. In particular, *Acetobacter* is able to grow in an acidic environment with a pH below 5.5 (Kersters et al., 2006) and the leaf cytoplasm of *Hamamelis* exhibits a pH of ∼4 (S. Sauer, unpubl. data). In addition to the prominent phylogenetic groups we further detected consistently other groups of lower abundances in the plant compartments in particular of *Achillea*, such as *Deinococcales*, *Firmicutes*, and their order *Lactobacillales*. This group has also been reported in other studies (Shade et al., 2013; Copeland et al., 2015; Hardoim et al., 2015; Pontonio et al., 2018; Massoni et al., 2020). *Lactobacillales* may have important and so far only little understood beneficial functions for plant health by producing secondary metabolites promoting plant growth and/or inhibiting growth of pathogens (Lamont et al., 2017; Pontonio et al., 2018; Daranas et al., 2019).

In contrast to bacteria, the fungal communities of both MPs exhibited pronounced differences. Whereas *Basidiomycota* dominated on leaves of *Achillea* during the early developmental stages and on flower buds and constituted 20–25% of total relative abundance on flowers, they comprised only minor proportions on leaves of *Hamamelis*. Interestingly, on flowers fungal taxa, which could not be further affiliated taxonomically, constituted >35%. This indicates that *Achillea* flowers harbor still unknown and obviously prominent fungi, which still need to be identified. Leaves of *Hamamelis* were greatly dominated by *Ascomycota*, as were the later leaf stages of *Achillea*. In soil, also *Ascomycota* were abundant but different orders than on *Hamamelis* leaves. The ascomycotal taxa detected on *Hamamelis* and *Achillea*, such as *Didymella*, *Microsphaerella*, *Cladosporium*, *Microcyclospora*, and *Uwebraunia*, are widespread among many different plants like *Cannabis*, wheat, sugar cane and olive tree (Hardoim et al., 2015; Grudzinska-Sterno et al., 2016; Brader et al., 2017; Gomes et al., 2018; Hamonts et al., 2018). These fungi include saprophytes but also a few potential pathogens (Peršoh, 2015; Jia et al., 2016; Thapa and Prasanna, 2018). The *Basidiomycota* in the *Achillea* microbiome consisted predominantly of yeasts such as *Leucosporidium* and *Filobasidium*, which were present on leaves during the earlier stages and later on flower buds and flowers. Basidiomycotal yeasts can reduce infection and sporulation of fungal pathogens (Elad et al., 1994) and thus may act as protectants for *Achillea*. Yeasts of both fungal phyla on flowers appear to be protective and beneficial for floral health by suppressing growth of pathogenic fungi and bacteria and by producing volatile organic compounds attracting pollinators, but are little studied so far (Klaps et al., 2020). The predominance of ascomycotal genera such as *Didymella* and *Septoria*, already reported for *Achillea* (Hatamzadeh et al., 2018, 2020), on the later leaf stages may, in fact, indicate a stronger impact of these pathogens on the aging leaves. *Basidiomycota* seem to be less widespread among plants and constitute lower fractions of fungal phyllosphere communities than *Ascomycota* (Jin et al., 2015; Grudzinska-Sterno et al., 2016; Cregger et al., 2018; Hamonts et al., 2018). Thus, there is a need to intensify studies on *Basidiomycota*, which appear to be more important interaction partners with other plant-associated microbes and the host plant than assumed previously (Martin et al., 2015; Sidorova and Voronina, 2019).

### Core Taxa of *Achillea*

The identification of core microbial communities of plants is critical to better understand the significance of the core taxa for the health of the host plant and its compartments and maybe of the functional roles and mutual interactions of these core taxa with the respective host plant (Shade and Handelsman, 2011). Microbial core communities of plants so far have been rarely investigated and their epi- and endophytic communities have never been distinguished. Hamonts et al. (2018) studied the core bacterial and fungal communities in the leaf, stalk and rhizosphere of sugar cane of different regions in Queensland, Australia. The bacterial core communities of switchgrass and miscanthus of different plots in an experimental area of biofuel crops in Michigan, United States, were studied by Grady et al. (2019) and bacterial core communities of apple flowers of six trees of the same cultivar in an agricultural research station in Wisconsin, United States (Shade et al., 2013). The bacterial core community of the phyllosphere of the Manuka tree (*Leptospermum scoparium*) from five different regions in New Zealand was studied by Noble et al. (2020).

We assessed the bacterial and fungal communities of *Achillea* at four different locations in summer when the plant was fully developed. Hence, we were able to identify the bacterial and fungal epi- and endophytic core communities. Our findings revealed that the epiphytic bacterial and fungal core communities were more diverse than the endophytic communities on leaves and flowers, except for fungi on leaves, even though there was a considerable overlap in the taxa of both communities. The core communities constituted a systematically much higher proportion, i.e., >75% of the total abundance as compared to the total number of taxa, and this difference was consistently higher for the endo- than for the epiphytic communities. A similar finding was reported for the bacterial core community of Manuka trees (Noble et al., 2020), for bacterial and fungal core communities of sugar cane (Hamonts et al., 2018), and switchgrass and miscanthus (Grady et al., 2019), thus, emphasizing the general significance of the microbial core communities for the plants. Our findings further imply, in agreement with previous work on mangrove ecosystems (Yao et al., 2019), that the highly abundant endophytic microbial core communities have a generally greater impact on the interactions with the host plant than the epiphytic core communities. The latter reveal a higher diversity, assuming that they are more affected by local effects such as transmission of location-specific taxa, weather and microclimatic conditions. The differences between the epi- and endophytic microbial core communities also indicate that our methodological separation of both communities was effective. However, we cannot rule out that it did not always remove all attached bacteria from the surfaces during the washing procedure and some overlap between both fractions might still occur.

The bacterial and fungal core communities comprised quite a few rare taxa, which were not among the most abundant ones. This suggests that these members of the rare phyllosphere do play a significant role in the mutual interactions among the microbes and with the host plant. This has been hypothesized for rare microbial taxa in a habitat context in general (Jousset et al., 2017) and emphasizes that these taxa should not be neglected in such investigations. Consequently, future work needs to elucidate their specific functions within the phyllosphere.

The bacterial core community comprised genera, which occurred on leaf and flower in the epi- as well as endophytic compartments including several *Actinobacteria*, *Deinococcus*, *Alpha*-, and *Gammaproteobacteria*. Some of these genera, such as *Erwinia*, *Sphingomonas*, *Deinococcus*, have also been reported as prominent genera of leaves of the Manuka tree, sugar cane, switchgrass and miscanthus and of flowers of apple tree (Shade et al., 2013; Hamonts et al., 2018; Grady et al., 2019; Noble et al., 2020). The fact that we detected core members occurring only in the epi- or endophytic leaf or flower community indicates that some taxa were distinctly adapted to one of these habitats. The flower-exclusive core community comprised primarily *Lactobacillales* and *Enterobacteriaceae* and some of them were exclusive to the epi- or endophytic compartment indicating their very specific adaptation to these habitats rich in easily degradable carbohydrates. One mode of transmission of the flower-associated taxa certainly was insect pollination as indicated by the flower-specific epiphytic *Gilliamella*, well known from the gut of bees, hornets and bumble bees (Moran, 2015; Graystock et al., 2017; Suenami et al., 2019; Zhang et al., 2020). Interestingly, we also detected *Gilliamella* on leaves of *Hamamelis* and on leaves of *Achillea* in spring, assuming a transmission by insect feces or resting insects, as both plants did not flower at this time.

Several fungal core taxa occurred both in the epi- and endophytic leaf and flower compartments. They affiliated to *Mycosphaerellaceae*, *Didymellaceae* and other ascomycotal families but also to basidiomycotal families such as *Sporidiobolaceae* and *Bulleraceae*. Quite a few fungal core genera were exclusive to leaves and four to the flower including the highly abundant phytopathogenic ascomycotal genus *Botryosphaeria* and three basidiomycotal genera, exclusive in the epiphytic flower. Only few of these core genera were reported in a comparable study by Hamonts et al. (2018) on the core fungal community of sugar cane, such as *Bullera*, even though most core taxa affiliated to orders also found in our study. Several fungi of the core community have been reported to inhibit growth of pathogenic microbes and to produce volatile organic compounds attracting pollinators such as *Aureobasidium* and *Sporobolomyces* (Golubev and Nakase, 1997; Caruso et al., 2020; Klaps et al., 2020).

As our study on the fungal and bacterial core communities of *Achillea* represents pioneering work, it is too early to draw conclusions on a more general significance of these core communities. Taxa of the core communities certainly are most important for the health of the host plant, in our study the MP *Achillea*, presumably including growth-promoting and protecting features but also by controlling potentially pathogenic microbes of the core communities. Further, our results show that it is worth distinguishing between epi- and endophytic communities as we found distinct differences between both communities.

### The Microbiome of *Achillea* and *Hamamelis* Under Medicinal Aspects and Outlook

Both MPs we studied are well known for various applications, and a variety of bioactive compounds have been identified in *Achillea* (Candan et al., 2003; Saeidnia et al., 2011; Dias et al., 2013; Hatamzadeh et al., 2020; Apel et al., 2021) and *Hamamelis* (Da Silva et al., 2000; Saénchez-Tena et al., 2012; Duckstein et al., 2012a; Rocasalbas et al., 2013). Most of these studies focused on the bioactive compounds and their potential application, whereas only one study assessed the chemical transformation by the natural microflora upon spontaneous fermentation (Duckstein et al., 2012a). In the present investigation we comprehensively characterized the epi- and endophytic bacterial and fungal microbiome of above-ground compartments of *Achillea* and of the leaves of *Hamamelis*. The results show distinct microbiomes of each MP and seasonal variations. Among the bacteria and fungi in particular of the endophytic compartments taxa were identified of which certain species are known to produce bioactive compounds including ones with antimicrobial and antioxidant activity, which appear to be important in establishing and maintaining the health of the host plant. These microbes interact with each other and the host plant, and it has been shown that such interactions may be instrumental for the production of bioactive compounds (Caruso et al., 2020). Hence, it is highly probable that the microbiome of both MPs investigated affects or may even be instrumental in shaping the bouquet of the bioactive compounds the MP produced and which are key to the medical treatment. The microbiome and/or single microbial taxa of both MPs presumably are also important in further processing these MP and their extracts, e.g., by fermentation as already shown for other MPs and their microbiomes (Schwarzenberger et al., 2012; Lorenz et al., 2013; Duckstein and Stintzing, 2015; Hussain et al., 2016). Thus, future research needs to elucidate the role of the microbiome in the production and conversion of host plant-specific bioactive compounds.

## Data Availability Statement

The bacterial 16S rRNA gene and fungal ITS amplicon sequencing data have been deposited in the European Nucleotide Archive (ENA) at EMBL-EBI under accession number PRJEB40947 (https://www.ebi.ac.uk/ena/browser/view/PRJEB40947).

## Author Contributions

FS, DK, SS, and MS designed the study. SS carried out the practical work, sample processing, and data analyses. LD carried out the bioinformatics and statistical analyses. SS and MS wrote the manuscript. All authors reviewed the manuscript and approved it for publication.

## Conflict of Interest

SS, DK, and FS were employed by company WALA Heilmittel GmbH. The remaining authors declare that the research was conducted in the absence of any commercial or financial relationships that could be construed as a potential conflict of interest.

## Footnotes

1 https://sourceforge.net/projects/bbmap/

## References

[B1] Abarenkov K. Zirk A. Piirmann T. Pöhönen R. Ivanov F. Nilsson R. H. (2020). *UNITE general FASTA release for eukaryotes 2.* London: UNITE Community, 10.15156/BIO/786354

[B2] Aleklett K. Hart M. Shade A. (2014). The microbial ecology of flowers: An emerging frontier in phyllosphere research 1. *Botany* 92 253–266. 10.1139/cjb-2013-0166 33356898

[B3] Ansary W. R. Prince F. R. K. Haque E. Sultana F. West H. M. Rahman M. (2018). Endophytic Bacillus spp. from medicinal plants inhibit mycelial growth of Sclerotinia sclerotiorum and promote plant growth. *Z. Naturforsch.* 73c 247–256. 10.1515/znc-2018-0002 29652669

[B4] Apel L. Lorenz P. Urban S. Sauer S. Spring O. Stintzing F. C. (2021). Phytochemical characterization of different yarrow species (Achillea sp.) and investigations into their antimicrobial activity. *Z. Naturforsch.* 76c 55–65. 10.1515/znc-2020-0149 32897872

[B5] Atar E. März C. Schnetger B. Wagner T. Aplin A. (2019). Local to global controls on the deposition of organic-rich muds across the late jurassic laurasian seaway. *J. Geol. Soc. London.* 176 1143–1153. 10.1144/jgs2019-031

[B6] Barman D. Dkhar M. S. (2020). Seasonal variation influence endophytic actinobacterial communities of medicinal plants from tropical deciduous forest of Meghalaya and characterization of their plant growth-promoting potentials. *Curr. Microbiol.* 77 1689–1698. 10.1007/s00284-020-01988-3 32300926

[B7] Benedek B. Kopp B. Melzig M. F. (2007). *Achillea millefolium* L. s.l. – Is the anti-inflammatory activity mediated by protease inhibition? *J. Ethnopharmacol.* 113 312–317. 10.1016/j.jep.2007.06.014 17689902

[B8] Berg G. Rybakova D. Grube M. Köberl M. (2016). The plant microbiome explored: Implications for experimental botany. *J. Exp. Bot.* 67 995–1002. 10.1093/jxb/erv466 26547794 PMC5395086

[B9] Bolger A. M. Lohse M. Usadel B. (2014). Trimmomatic: A flexible trimmer for Illumina sequence data. *Bioinformatics* 30 2114–2120. 10.1093/bioinformatics/btu170 24695404 PMC4103590

[B10] Brader G. Compant S. Vescio K. Mitter B. Trognitz F. Ma L. J. (2017). Ecology and genomic insights into plant-pathogenic and plant-nonpathogenic endophytes. *Annu. Rev. Phytopathol.* 55 61–83. 10.1146/annurev-phyto-080516-035641 28489497

[B11] Bray J. R. Curtis J. T. (1957). An ordination of the upland forest communities of Southern Wisconsin. *Ecol. Monogr.* 27 325–349. 10.2307/1942268

[B12] Candan F. Unlu M. Tepe B. Daferera D. Polissiou M. Sökmen A. (2003). Antioxidant and antimicrobial activity of the essential oil and methanol extracts of *Achillea millefolium* subsp. millefolium Afan. (Asteraceae). *J. Ethnopharmacol.* 87 215–220. 10.1016/S0378-8741(03)00149-1 12860311

[B13] Cardinale M. Grube M. Erlacher A. Quehenberger J. Berg G. (2015). Bacterial networks and co-occurrence relationships in the lettuce root microbiota. *Environ. Microbiol.* 17 239–252. 10.1111/1462-2920.12686 25367329

[B14] Caruso G. Abdelhamid M. Kalisz A. Sekara A. (2020). Linking endophytic fungi to medicinal plants therapeutic activity. A case study on Asteraceae. *Agriculture* 10 286. 10.3390/agriculture10070286

[B15] Castronovo L. M. Calonico C. Ascrizzi R. Del Duca S. Delfino V. Chioccioli S. (2020). The cultivable bacterial microbiota associated to the medicinal plant *Origanum vulgare* L.: From antibiotic resistance to growth-inhibitory properties. *Front. Microbiol.* 11:862. 10.3389/fmicb.2020.00862 32457726 PMC7226918

[B16] Chen H. Wu H. Yan B. Zhao H. Liu F. Zhang H. (2018). Core microbiome of medicinal plant *Salvia miltiorrhiza* seed: A rich reservoir of beneficial microbes for secondary metabolism? *Int. J. Mol. Sci.* 19 672. 10.3390/ijms19030672 29495531 PMC5877533

[B17] Chen Y. Guo Q. Zhu Z. Zhang L. (2012). Changes in bioactive components related to the harvest time from the spicas of Prunella vulgaris. *Pharm. Biol.* 50 1118–1122. 10.3109/13880209.2012.658477 22686260

[B18] Comeau D. Novinscak A. Joly D. L. Filion M. (2020). Spatio-temporal and cultivar-dependent variations in the Cannabis microbiome. *Front. Microbiol.* 11:491. 10.3389/fmicb.2020.00491 32265895 PMC7105690

[B19] Copeland J. K. Yuan L. Layeghifard M. Wang P. W. Guttman D. S. (2015). Seasonal community succession of the phyllosphere microbiome. *Mol. Plant-Microbe Interact.* 28 274–285. 10.1094/MPMI-10-14-0331-FI 25679538

[B20] Cregger M. A. Veach A. M. Yang Z. K. Crouch M. J. Vilgalys R. Tuskan G. A. (2018). The Populus holobiont: Dissecting the effects of plant niches and genotype on the microbiome. *Microbiome* 6 31. 10.1186/s40168-018-0413-8 29433554 PMC5810025

[B21] Da Silva A. P. Rocha R. Silva C. M. L. Mira L. Filomena Duarte M. Helena Florncio M. (2000). Antioxidants in medicinal plant extracts. A research study of the antioxidant capacity of Crataegus, Hamamelis and Hydrastis. *Phyther. Res.* 14 612–616. 10.1002/1099-1573(200012)14:8<612::aid-ptr677>3.0.co;2-t 11113998

[B22] Daranas N. Roselló G. Cabrefiga J. Donati I. Francés J. Badosa E. (2019). Biological control of bacterial plant diseases with Lactobacillus plantarum strains selected for their broad-spectrum activity. *Ann. Appl. Biol.* 174 92–105. 10.1111/aab.12476 30686827 PMC6334523

[B23] Dias M. I. Barros L. Dueñas M. Pereira E. Carvalho A. M. Alves R. C. (2013). Chemical composition of wild and commercial *Achillea millefolium* L. and bioactivity of the methanolic extract, infusion and decoction. *Food Chem.* 141 4152–4160. 10.1016/j.foodchem.2013.07.018 23993599

[B24] Duckstein S. M. Lorenz P. Stintzing F. C. (2012a). Conversion of phenolic constituents in aqueous *Hamamelis virginiana* leaf extracts during fermentation. *Phytochem. Anal.* 23 588–597. 10.1002/pca.2359 22434718

[B25] Duckstein S. M. Lotter E. M. Meyer U. Lindequist U. Stintzing F. C. (2012b). Phenolic constituents from Alchemilla vulgaris L. and Alchemilla mollis (Buser) Rothm. at different dates of harvest. *Z. Naturforsch.* 67c 529–540. 23413745

[B26] Duckstein S. M. Stintzing F. C. (2015). LC – MS^n^ characterization of steroidal saponins in Helleborus niger L. roots and their conversion products during fermentation. *Steroids* 93 47–59. 10.1016/j.steroids.2014.09.011 25449769

[B27] Duda S. C. Mărghitaş L. A. Dezmirean D. Duda M. Mărgăoan R. Bobiş O. (2015). Changes in major bioactive compounds with antioxidant activity of Agastache foeniculum, Lavandula angustifolia, Melissa officinalis and Nepeta cataria: Effect of harvest time and plant species. *Ind. Crops Prod.* 77 499–507. 10.1016/j.indcrop.2015.09.045

[B28] Edgar R. C. (2010). Search and clustering orders of magnitude faster than BLAST. *Bioinformatics* 26 2460–2461. 10.1093/bioinformatics/btq461 20709691

[B29] Elad Y. Köhl J. Fokkema N. J. (1994). Control of infection and sporulation of Botrytis cinerea on bean and tomato by saprophytic yeasts. *Phytopathology* 100 315–336. 10.1094/Phyto-84-1193

[B30] EMA/HMPC (2018). *Addendum to assessment report on Achillea millefolium L., flos.* London: Committee On Herbal Medicinal Products (HMPC).

[B31] EMEA (2009). *Committee on herbal medicinal products (HMPC) assessment. Assessment report on Hamamelis virginiana L., cortex Hamamelis virginiana L., folium Hamamelis virginiana L., folium et cortex aut ramunculus destillatum.* London: EMEA.

[B32] Golinska P. Wypij M. Agarkar G. Rathod D. Dahm H. Rai M. (2015). Endophytic actinobacteria of medicinal plants: Diversity and bioactivity. *Antonie van Leeuwenhoek Int. J. Gen. Mol. Microbiol.* 108 267–289. 10.1007/s10482-015-0502-7 26093915 PMC4491368

[B33] Golubev W. Nakase T. (1997). Mycocinogeny in the genus Bullera: Taxonomic specificity of sensitivity to the mycocin produced by Bullera sinensis. *FEMS Microbiol. Lett.* 146 59–64. 10.1016/S0378-1097(96)00450-8 8997707

[B34] Gomes T. Pereira J. A. Benhadi J. Lino-Neto T. Baptista P. (2018). Endophytic and epiphytic phyllosphere fungal communities are shaped by different environmental factors in a Mediterranean ecosystem. *Microb. Ecol.* 76 668–679. 10.1007/s00248-018-1161-9 29500493

[B35] Grady K. L. Sorensen J. W. Stopnisek N. Guittar J. Shade A. (2019). Assembly and seasonality of core phyllosphere microbiota on perennial biofuel crops. *Nat. Commun.* 10 4135. 10.1038/s41467-019-11974-4 31515535 PMC6742659

[B36] Graystock P. Rehan S. M. McFrederick Q. S. (2017). Hunting for healthy microbiomes: determining the core microbiomes of Ceratina, Megalopta, and Apis bees and how they associate with microbes in bee collected pollen. *Conserv. Genet.* 18 701–711. 10.1007/s10592-017-0937-7

[B37] Grudzinska-Sterno M. Yuen J. Stenlid J. Djurle A. (2016). Fungal communities in organically grown winter wheat affected by plant organ and development stage. *Eur. J. Plant Pathol.* 146 401–417. 10.1007/s10658-016-0927-5

[B38] Gupta S. Chaturvedi P. Kulkarni M. G. Van Staden J. (2020). A critical review on exploiting the pharmaceutical potential of plant endophytic fungi. *Biotechnol. Adv.* 39 107462. 10.1016/j.biotechadv.2019.107462 31669137

[B39] Hamonts K. Trivedi P. Garg A. Janitz C. Grinyer J. Holford P. (2018). Field study reveals core plant microbiota and relative importance of their drivers. *Environ. Microbiol.* 20 124–140. 10.1111/1462-2920.14031 29266641

[B40] Hardoim P. R. van Overbeek L. S. Berg G. Pirttilä A. M. Compant S. Campisano A. (2015). The hidden world within plants: Ecological and evolutionary considerations for defining functioning of microbial endophytes. *Microbiol. Mol. Biol. Rev.* 79 293–320. 10.1128/mmbr.00050-14 26136581 PMC4488371

[B41] Haron M. H. Tyler H. L. Chandra S. Moraes R. M. Jackson C. R. Pugh N. D. (2019). Plant microbiome-dependent immune enhancing action of *Echinacea purpurea* is enhanced by soil organic matter content. *Sci. Rep.* 9 136. 10.1038/s41598-018-36907-x 30644442 PMC6333828

[B42] Harrison J. G. Griffin E. A. (2020). The diversity and distribution of endophytes across biomes, plant phylogeny and host tissues: how far have we come and where do we go from here? *Environ. Microbiol.* 22 2107–2123. 10.1111/1462-2920.14968 32115818 PMC7679042

[B43] Hatamzadeh S. Rahnama K. Nasrollahnejad S. Fotouhifar K.-B. Hemmati K. White J. F. (2018). Septoria malagutii as an endophytic fungus of *Achillea millefolium* from Iran. *Mycol. Iran.* 5 105–107. 10.22043/MI.2018.120384

[B44] Hatamzadeh S. Rahnama K. Nasrollahnejad S. Fotouhifar K. B. Hemmati K. White J. F. (2020). Isolation and identification of L-asparaginase-producing endophytic fungi from the Asteraceae family plant species of Iran. *PeerJ* 2020 1–16. 10.7717/peerj.8309 31976175 PMC6968492

[B45] Huang W. Long C. Lam E. (2018). Roles of plant-associated microbiota in traditional herbal medicine. *Trends Plant Sci.* 23 559–562. 10.1016/j.tplants.2018.05.003 29802067

[B46] Hussain A. Bose S. Wang J. H. Yadav M. K. Mahajan G. B. Kim H. (2016). Fermentation, a feasible strategy for enhancing bioactivity of herbal medicines. *Food Res. Int.* 81 1–16. 10.1016/j.foodres.2015.12.026

[B47] Ivanova N. V. Kuzmina M. L. Braukmann T. W. A. Borisenko A. V. Zakharov E. V. (2016). Authentication of herbal supplements using next-generation sequencing. *PLoS One* 11:e0156426. 10.1371/journal.pone.0156426 27227830 PMC4882080

[B48] Jia M. Chen L. Xin H. L. Zheng C. J. Rahman K. Han T. (2016). A friendly relationship between endophytic fungi and medicinal plants: A systematic review. *Front. Microbiol.* 7:906. 10.3389/fmicb.2016.00906 27375610 PMC4899461

[B49] Jin H. Yang X. Lu D. Li C. Yan Z. Li X. (2015). Phylogenic diversity and tissue specificity of fungal endophytes associated with the pharmaceutical plant, Stellera chamaejasme L. revealed by a cultivation-independent approach. *Antonie van Leeuwenhoek, Int. J. Gen. Mol. Microbiol.* 108 835–850. 10.1007/s10482-015-0538-8 26194722

[B50] Jones M. S. Fu Z. Reganold J. P. Karp D. S. Besser T. E. Tylianakis J. M. (2019). Organic farming promotes biotic resistance to foodborne human pathogens. *J. Appl. Ecol.* 56 1117–1127. 10.1111/1365-2664.13365

[B51] Jost L. (2006). Entropy and diversity – Jost – 2006 – Oikos – Wiley Online Library. *Oikos* 113 363–375. 10.1111/j.2006.0030-1299.14714.x/full

[B52] Jousset A. Bienhold C. Chatzinotas A. Gallien L. Gobet A. Kurm V. (2017). Where less may be more: How the rare biosphere pulls ecosystems strings. *ISME J.* 11 853–862. 10.1038/ismej.2016.174 28072420 PMC5364357

[B53] Junker R. R. Keller A. (2015). Microhabitat heterogeneity across leaves and flower organs promotes bacterial diversity. *FEMS Microbiol. Ecol.* 91 fiv097. 10.1093/femsec/fiv097 26253507

[B54] Kembel S. W. Cowan P. D. Helmus M. R. Cornwell W. K. Morlon H. Ackerly D. D. (2010). Picante: R tools for integrating phylogenies and ecology. *Bioinformatics* 26 1463–1464. 10.1093/bioinformatics/btq166 20395285

[B55] Kersters K. Lisdiyanti P. Komagata K. Swings J. (2006). “The family Acetobacteraceae: The genera Acetobacter, Acidomonas, Asaia, Gluconacetobacter, Gluconobacter, and Kozakia,” in *The Prokaryotes*, eds Dworkin M. Falkow S. Rosenberg E. Schleifer K. H. Stackebrandt E. (New York, NY: Springer), 10.1007/0-387-30745-1_9

[B56] Klaps J. Lievens B. Álvarez-Pérez S. (2020). Towards a better understanding of the role of nectar-inhabiting yeasts in plant-animal interactions. *Fungal Biol. Biotechnol.* 7 1. 10.1186/s40694-019-0091-8 31921433 PMC6947986

[B57] Köberl M. Erschen S. Etemadi M. White R. A. El-Arabi T. F. Berg G. (2019). Deciphering the microbiome shift during fermentation of medicinal plants. *Sci. Rep.* 9 13461. 10.1038/s41598-019-49799-2 31530872 PMC6748931

[B58] Köberl M. Schmidt R. Ramadan E. M. Bauer R. Berg G. (2013). The microbiome of medicinal plants: Diversity and importance for plant growth, quality, and health. *Front. Microbiol.* 4:400. 10.3389/fmicb.2013.00400 24391634 PMC3868918

[B59] Köngül E. Taş Ö Paşayeva L. Karatoprak G. Ş (2017). Analysis of the cytotoxic effects of *Achillea millefolium* L. extracts on MCF7 cell line. *Proceedings* 1 1077. 10.3390/proceedings1101077

[B60] Lamont J. R. Wilkins O. Bywater-Ekegärd M. Smith D. L. (2017). From yogurt to yield: Potential applications of lactic acid bacteria in plant production. *Soil Biol. Biochem.* 111 1–9. 10.1016/j.soilbio.2017.03.015

[B61] Leff J. W. Del Tredici P. Friedman W. E. Fierer N. (2015). Spatial structuring of bacterial communities within individual Ginkgo biloba trees. *Environ. Microbiol.* 17 2352–2361. 10.1111/1462-2920.12695 25367625

[B62] Lorenz P. Conrad J. Stintzing F. C. (2013). Metabolic fate of depsides and alkaloid constituents in aqueous extracts from Mercurialis perennis L. during fermentation. *Chem. Biodivers.* 10 1706–1723. 24078603 10.1002/cbdv.201200424

[B63] Lumactud R. Fulthorpe R. R. (2018). Endophytic bacterial community structure and function of herbaceous plants from petroleum hydrocarbon contaminated and non-contaminated sites. *Front. Microbiol.* 9:1926. 10.3389/fmicb.2018.01926 30190710 PMC6115521

[B64] Lumactud R. Shen S. Y. Lau M. Fulthorpe R. (2016). Bacterial endophytes isolated from plants in natural oil seep soils with chronic hydrocarbon contamination. *Front. Microbiol.* 7:755. 10.3389/fmicb.2016.00755 27252685 PMC4878295

[B65] Lyu D. Backer R. Subramanian S. Smith D. L. (2020). Phytomicrobiome coordination signals hold potential for climate change-resilient agriculture. *Front. Plant Sci.* 11:634. 10.3389/fpls.2020.00634 32523595 PMC7261841

[B66] Maggini V. De Leo M. Mengoni A. Gallo E. R. Miceli E. Reidel R. V. B. (2017). Plant-endophytes interaction influences the secondary metabolism in *Echinacea purpurea* (L.) Moench: An *in vitro* model. *Sci. Rep.* 7 16924. 10.1038/s41598-017-17110-w 29208923 PMC5717142

[B67] Martin R. Gazis R. Skaltsas D. Chaverri P. Hibbett D. (2015). Unexpected diversity of basidiomycetous endophytes in sapwood and leaves of Hevea. *Mycologia* 107 284–297. 10.3852/14-206 25572095

[B68] Massoni J. Bortfeld-Miller M. Jardillier L. Salazar G. Sunagawa S. Vorholt J. A. (2020). Consistent host and organ occupancy of phyllosphere bacteria in a community of wild herbaceous plant species. *ISME J.* 14 245–258. 10.1038/s41396-019-0531-8 31624344 PMC6908658

[B69] Moran N. A. (2015). Genomics of the honey bee microbiome. *Curr. Opin. Insect Sci.* 10 22–28. 10.1016/j.cois.2015.04.003 26140264 PMC4484875

[B70] Müller D. B. Vogel C. Bai Y. Vorholt J. A. (2016). The plant microbiota: Systems-level insights and perspectives. *Annu. Rev. Genet.* 50 211–234. 10.1146/annurev-genet-120215-034952 27648643

[B71] Naik K. Mishra S. Srichandan H. Singh P. K. Sarangi P. K. (2019). Plant growth promoting microbes: Potential link to sustainable agriculture and environment. *Biocatal. Agric. Biotechnol.* 21 101326. 10.1016/j.bcab.2019.101326

[B72] Noble A. S. Noe S. Clearwater M. J. Lee C. K. (2020). A core phyllosphere microbiome exists across distant populations of a tree species indigenous to New Zealand. *PLoS One* 15:e0237079. 10.1371/journal.pone.0237079 32790769 PMC7425925

[B73] Oksanen J. Blanchet F. G. Friendly M. Kindt R. Legendre P. McGlinn D. (2017). *vegan: Community Ecology Package. R package version 2.4−3.* Available online at: https://CRAN.R-project.org/package=vegan.

[B74] Ottesen A. R. González Peña A. White J. R. Pettengill J. B. Li C. Allard S. (2013). Baseline survey of the anatomical microbial ecology of an important food plant: Solanum lycopersicum (tomato). *BMC Microbiol.* 13:114. 10.1186/1471-2180-13-114 23705801 PMC3680157

[B75] Pacifico D. Squartini A. Crucitti D. Barizza E. Lo Schiavo F. Muresu R. (2019). The role of the endophytic microbiome in the grapevine response to environmental triggers. *Front. Plant Sci.* 10:1256. 10.3389/fpls.2019.01256 31649712 PMC6794716

[B76] Paradis E. Schliep K. (2018). Ape 5.0: An environment for modern phylogenetics and evolutionary analyses in R. *Bioinformatics* 35 526–528. 10.1093/bioinformatics/bty633 30016406

[B77] Parks D. H. Chuvochina M. Waite D. W. Rinke C. Skarshewski A. Chaumeil P. A. (2018). A standardized bacterial taxonomy based on genome phylogeny substantially revises the tree of life. *Nat. Biotechnol.* 36 996–1004. 10.1038/nbt.4229 30148503

[B78] Pereira J. M. Peixoto V. Teixeira A. Sousa D. Barros L. Ferreira I. C. F. R. (2018). *Achillea millefolium* L. hydroethanolic extract has phenolic compounds and inhibits the growth of human tumor cell lines. *Free Radic. Biol. Med.* 120 145. 10.1016/j.freeradbiomed.2018.04.477 29883784

[B79] Peršoh D. (2015). Plant-associated fungal communities in the light of meta’omics. *Fungal Divers.* 75 1–25. 10.1007/s13225-015-0334-9

[B80] Pontonio E. Di Cagno R. Tarraf W. Filannino P. De Mastro G. Gobbetti M. (2018). Dynamic and assembly of epiphyte and endophyte lactic acid bacteria during the life cycle of *Origanum vulgare* L. *Front. Microbiol.* 9:1372. 10.3389/fmicb.2018.01372 29997592 PMC6029521

[B81] R Core Team (2018). *R: A language and environment for statistical computing.* Vienna: R Foundation for Statistical Computing.

[B82] Rahman L. Shinwari Z. K. Iqrar I. Rahman L. Tanveer F. (2017). An assessment on the role of endophytic microbes in the therapeutic potential of Fagonia indica. *Ann. Clin. Microbiol. Antimicrob.* 16 53. 10.1186/s12941-017-0228-7 28764775 PMC5540543

[B83] Rehill B. J. Schultz J. C. (2012). Hormaphis hamamelidis fundatrices benefit by manipulating phenolic metabolism of their host. *J. Chem. Ecol.* 38 496–498. 10.1007/s10886-012-0115-9 22532245

[B84] Rillig M. C. Lehmann A. Lehmann J. Camenzind T. Rauh C. (2018). Soil biodiversity effects from field to fork. *Trends Plant Sci.* 23 17–24. 10.1016/j.tplants.2017.10.003 29146430

[B85] Ritz C. Baty F. Streibig J. C. Gerhard D. (2015). Dose-response analysis using R. *PLoS One* 10:e0146021. 10.1371/journal.pone.0146021 26717316 PMC4696819

[B86] Rocasalbas G. Francesko A. Touriño S. Fernández-Francos X. Guebitz G. M. Tzanov T. (2013). Laccase-assisted formation of bioactive chitosan/gelatin hydrogel stabilized with plant polyphenols. *Carbohydr. Polym.* 92 989–996. 10.1016/j.carbpol.2012.10.045 23399119

[B87] Rodriguez R. J. White J. F. Arnold A. E. Redman R. S. (2009). Fungal endophytes: Diversity and functional roles: Tansley review. *New Phytol.* 182 314–330. 10.1111/j.1469-8137.2009.02773.x 19236579

[B88] Saeidnia S. Gohari A. R. Mokhber-Dezfuli N. Kiuchi F. (2011). A review on phytochemistry and medicinal properties of the genus Achillea. *DARU, J. Pharm. Sci.* 19 173–186. PMC3232110 22615655

[B89] Saénchez-Tena S. Fernaéndez-Cachoén M. L. Carreras A. Mateos-Martién M. L. Costoya N. Moyer M. P. (2012). Hamamelitannin from witch hazel (*Hamamelis virginiana*) displays specific cytotoxic activity against colon cancer cells. *J. Nat. Prod.* 75 26–33. 10.1021/np200426k 22216935

[B90] Satari A. H. Zargar M. I. Dharne M. Bansal R. (2016). Isolation and screening of endophytic fungi from *Achillea millefolium* L – a medicinal plant of Western Himalayas. *Imp. J. Interdiscip. Res.* 2 498–502.

[B91] Schmidt R. Köberl M. Mostafa A. Ramadan E. M. Monschein M. Jensen K. B. (2014). Effects of bacterial inoculants on the indigenous microbiome and secondary metabolites of chamomile plants. *Front. Microbiol.* 5:64. 10.3389/fmicb.2014.00064 24600444 PMC3928675

[B92] Schwarzenberger M. Stintzing F. Meyer U. Lindequist U. (2012). Biochemical, microbiological and phytochemical studies on aqueous-fermented extracts from Atropa belladonna L. Part 2 – Phytochemistry. *Pharmazie* 67 460–466. 10.1691/ph.2012.1651 22764583

[B93] Shade A. Handelsman J. (2011). Beyond the venn diagram: The hunt for a core microbiome. *Environ. Microbiol.* 14 4–12. 10.1111/j.1462-2920.2011.02585.x 22004523

[B94] Shade A. Patricia S. McManus P. S. Handelsmana J. (2013). Unexpected diversity during community succession in the apple flower microbiome. *MBio* 4 e602–e612. 10.1128/mBio.00602-12 23443006 PMC3585449

[B95] Sidorova I. Voronina E. (2019). “Bioactive secondary metabolites of basidiomycetes and its potential for agricultural plant growth promotion,” in *Secondary Metabolites of Plant Growth Promoting Rhizomicroorganisms: Discovery and Applications*, eds García-Estrada C. Singh H. B. Keswani C. Reddy M. S. Royano E. S. (Singapore: Springer), 3–26. 10.1007/978-981-13-5862-3_1

[B96] Suenami S. Konishi Nobu M. Miyazaki R. (2019). Community analysis of gut microbiota in hornets, the largest eusocial wasps, Vespa mandarinia and V. simillima. *Sci. Rep.* 9 9830. 10.1038/s41598-019-46388-1 31285515 PMC6614390

[B97] Sun J. Chang M. Li H. Zhang Z. Chen Q. Chen Y. (2019). Endophytic bacteria as contributors to theanine production in *Camellia sinensis*. *J. Agric. Food Chem.* 67 10685–10693. 10.1021/acs.jafc.9b03946 31479251

[B98] Taghinasab M. Jabaji S. (2020). Cannabis microbiome and the role of endophytes in modulating the production of secondary metabolites: An overview. *Microorganisms* 8 355. 10.3390/microorganisms8030355 32131457 PMC7143057

[B99] Teimoori-Boghsani Y. Ganjeali A. Cernava T. Müller H. Asili J. Berg G. (2020). Endophytic fungi of native Salvia abrotanoides plants reveal high taxonomic diversity and unique profiles of secondary metabolites. *Front. Microbiol.* 10:3013. 10.3389/fmicb.2019.03013 32010087 PMC6978743

[B100] Thapa S. Prasanna R. (2018). Prospecting the characteristics and significance of the phyllosphere microbiome. *Ann. Microbiol.* 68 229–245. 10.1007/s13213-018-1331-5

[B101] Toju H. Peay K. G. Yamamichi M. Narisawa K. Hiruma K. Naito K. (2018). Core microbiomes for sustainable agroecosystems. *Nature Plants* 4 247–257. 10.1038/s41477-018-0139-4 29725101

[B102] Uroz S. Courty P. E. Oger P. (2019). Plant symbionts are engineers of the plant-associated microbiome. *Trends Plant Sci.* 24 905–916. 10.1016/j.tplants.2019.06.008 31288964

[B103] Vokou D. Genitsaris S. Karamanoli K. Vareli K. Zachari M. Voggoli D. (2019). Metagenomic characterization reveals pronounced seasonality in the diversity and structure of the phyllosphere bacterial community in a mediterranean ecosystem. *Microorganisms* 7 518. 10.3390/microorganisms7110518 31683878 PMC6920919

[B104] Vurukonda S. S. K. P. Giovanardi D. Stefani E. (2018). Plant growth promoting and biocontrol activity of Streptomyces spp. as endophytes. *Int. J. Mol. Sci.* 19 952. 10.3390/ijms19040952 29565834 PMC5979581

[B105] Wei N. Ashman T. L. (2018). The effects of host species and sexual dimorphism differ among root, leaf and flower microbiomes of wild strawberries *in situ*. *Sci. Rep.* 8 5195. 10.1038/s41598-018-23518-9 29581521 PMC5979953

[B106] Yao H. Sun X. He C. Maitra P. Li X. C. Guo L. D. (2019). Phyllosphere epiphytic and endophytic fungal community and network structures differ in a tropical mangrove ecosystem. *Microbiome* 7 57. 10.1186/s40168-019-0671-0 30967154 PMC6456958

[B107] Zhang Z. J. Huang M. F. Qiu L. F. Song R. H. Zhang Z. X. Ding Y. W. (2020). Diversity and functional analysis of Chinese bumblebee gut microbiota reveal the metabolic niche and antibiotic resistance variation of Gilliamella. *Insect Sci.* 28 302–314. 10.1111/1744-7917.12770 32101381

