# Supplementary material for: The Microbiome of the Medicinal Plants Achillea millefolium L. and Hamamelis virginiana L
Source: Front Microbiol. 2021 Jul 20;12:696398. doi: 10.3389/fmicb.2021.696398 (PMC8329415; doi:10.3389/fmicb.2021.696398)
Supplement: Supplementary file 1 [file Presentation_1.pdf]

The microbiome of the medicinal plants *Achillea millefolium* L. *and* *Hamamelis virginiana* L.

Simon Sauer[1,2], Leon Dlugosh[2], Dietmar R. Kammerer[1], Florian C. Stintzing[1*], Meinhard Simon[2*]

[1]WALA Heilmittel GmbH, Dorfstrasse 1, 73087 Bad Boll, Germany

[2] Institute for Chemistry and Biology of the Marine Environment, University of Oldenburg, Carl von Ossietzky Strasse 9-11, 26129 Oldenburg, Germany

*** Correspondence:**

Florian Stintzing
florian.stintzing@wala.de

Meinhard Simon
m.simon@icbm.de

Supplementary material

Table S1 Statistical comparison of the mean values of richness and effective number (EN) diversity of the epi- and endophytic bacterial and fungal leaf and flower and soil communities of all samples.

Table S2 The core microbiome of *Achillea* of four locations in southern Germany and Switzerland.

Table S3 Taxonomy of core bacteria of the total plant, total leaf, epi- and endophytic leaf and flower communities of *Achillea* not detected in the soil core microbiome.

Table S4 Taxonomy of core fungi of the total plant, total leaf, epi- and endophytic leaf and flower communities of *Achillea* not detected in the soil core microbiome.

Figure S1 Rarefaction curves of bacterial and fungal species.

Figure S2 PERMANOVA of bacterial and fungal plant samples of the time-series of *Achillea* and *Hamamelis.*

Figure S3 Metal and oxide constituents of the soil of locations 1 to 4, where *Achillea* samples were collected for the core microbiome assessment.

Figure S4 NMDS analysis of bacterial and fungal communities in the soils of locations 1 to 4, where *Achillea* samples were collected for the core microbiome assessment.

Figure S5 Heat map and cluster analysis of the 40 most abundant bacterial species and fungal genera in the triplicate soil samples at locations 1 to 4, where *Achillea* samples were collected for the core microbiome assessment.

Figure S6 Heat map and cluster analysis of the 40 most abundant bacterial species and fungal genera in the replicate samples of the epi- and endophytic floral compartments of *Achillea* at locations 1 to 4, where samples were collected for the core microbiome assessment.

Figure S7 Heat map and cluster analysis of the 40 most abundant bacterial species and fungal genera in the replicate samples of the epi- and endophytic floral compartments of *Achillea* at locations 1 to 4, where samples were collected for the core microbiome assessment.

Figure S8 Heat map of the seasonal dynamics of the 50 most abundant bacterial genera of the epi- and endophytic leaf, bud and flower and the soil communities of *Achillea* and of the epi- and endophytic leaf and the soil communities of *Hamamelis.*

Figure S9 Heat map of the seasonal dynamics of the 50 most abundant fungal genera of the epi- and endophytic leaf, bud and flower and the soil communities of *Achillea* and of the epi- and endophytic leaf and the soil communities of *Hamamelis*.

TABLE S1 I p-values of the statistical comparison of the mean values of richness and effective number (EN) diversity of the epi- and endophytic bacterial and fungal leaf and flower and soil communities of all samples determined by ANOVA and post-hoc test. Color codes give p-values <0.01 (green), 0.01-0.05 (yellow) and >0.05 (red).

Bacteria Richness p-value

	Flower endo	Flower epi	Leaf endo	Leaf epi
Flower epi	0.0112			
Leaf endo	< 0.0000	0.0014		
Leaf epi	< 0.0000	0.0010	0.0004	
Soil	< 0.0000	< 0.0000	< 0.0000	< 0.0000

Fungi Richness p-value

	Flower endo	Flower epi	Leaf endo	Leaf epi
Flower epi	0.3749			
Leaf endo	0.0029	0.5527		
Leaf epi	< 0.0000	0.0006	0.0065	
Soil	< 0.0000	< 0.0000	< 0.0000	< 0.0000

Bacteria EN p-value

	Flower endo	Flower epi	Leaf endo	Leaf epi
Flower epi	0.0742			
Leaf endo	< 0.0000	0.0053		
Leaf epi	< 0.0000	0.0028	0.0327	
Soil	< 0.0000	< 0.0000	< 0.0000	< 0.0000

Fungi EN p-value

	Flower endo	Flower epi	Leaf endo	Leaf epi
Flower epi	0.1182			
Leaf endo	0.0011	0.0299		
Leaf epi	< 0.0000	0.0004	0.4153	
Soil	< 0.0000	< 0.0000	< 0.0000	< 0.0000

TABLE S2 l The core microbiome of *Achillea* of four locations in southern Germany and Switzerland (for location details see Table 1). Data are given on the basis of bacterial species and fungal genera for the compartments soil, total plant, leaf, flower and epi- and endophytic leaf and flower communities: numbers of total taxa, accumulated number of unique taxa of one location, mean of unique taxa at one location as percent of total abundance of all taxa at one location, number of core taxa (occurred in at least two triplicates or when more replicates than triplicates were used >50% of triplicates were considered), core taxa as percent of total taxa and of total abundance, number of compartment-specific core taxa and percent of total abundance, number of core taxa of total plant or plant compartment not detected in the soil core taxa or never detected in any single soil replicate and respective percentages of total abundance. n: absolute number.

Compartment	total taxa	Sum of unique taxa of one location	unique taxa at one location %	Core taxa			compartment specific core		plant core not in soil core		plant core never in soil	
				n	% of total taxa	% of total abundance	n	% of total abundance	n	% of total abundance	n	% of total abundance
**Bacteria species**												
Soil	1985	465	0.7	836	42.1	93.7	749	72.5	0	0	0	0
Plant	324	144	14.4	59	18.2	56.9	0	0	42	39.0	1	0.1
Leaf	511	183	5.0	129	25.2	83.8	0	0	88	56.3	6	0.6
Leaf_epi	913	334	2.2	230	25.2	80.5	53	1.5	146	48.0	9	0.6
Leaf_endo	659	307	2.4	113	17.1	90.1	2	0.8	79	66.6	8	1.0
Flower	344	163	6.5	37	10.8	50.2	0	0	31	41.2	2	0.4
Flower_epi	1002	612	9.0	80	8.0	54.2	9	1.9	59	40.3	5	1.0
Flower_endo	329	163	1.9	34	10.3	64.9	7	13.0	29	59.7	6	0.4
**Bacteria genus**												
Soil	826	150	0.2	438	53.0	97.2	346	58.7	0	0	0	0
Plant	166	61	9.3	45	27.1	73.6	0	0	14	12.8	1	0
Leaf	221	77	4.3	72	32.6	92.2	0	0	20	9.9	3	0.2
Leaf_epi	409	133	0.3	120	29.3	91.0	12	0.4	34	8.8	4	0.2
Leaf_endo	263	103	1.9	59	22.4	94.6	0	0	16	11.3	2	0.1
Flower	184	78	2.6	30	16.3	68.4	0	0	17	25.2	1	0.0
Flower_epi	451	236	1.8	73	16.2	73.7	7	3.4	29	22.4	4	0.2
Flower_endo	170	77	0.8	28	16.5	76.1	5	13.1	18	39.1	3	0.2
**Fungi genus**												
Soil	292	111	4.4	64	21.9	65.4	48	46.4	0	0	0	0
Plant	98	41	3.9	22	22.4	74.6	0	0	14	13.1	2	0.4
Leaf	132	43	5.1	37	28.0	89.5	0	0	26	21.7	3	0.6
Leaf_epi	160	52	1.8	40	25.0	91.5	9	0.9	30	22.0	5	1.0
Leaf_endo	163	64	5.4	42	25.8	88.5	5	1	29	23.2	4	0.6
Flower	110	59	1.4	21	19.1	89.8	0	0	13	31.9	2	0.3
Flower_epi	213	112	1.0	30	14.1	86.5	3	0.2	18	17.0	2	0.4
Flower_endo	104	56	1.6	19	18.3	94.6	0	0.0	11	47.1	1	0.3

TABLE S3 I Taxonomy of core bacteria of the total plant, total leaf, epi- and endophytic leaf and flower communities of *Achillea* not detected in the soil core microbiome. Occurrence in different plant compartments is marked with x. To determine core and specific plant tissue community, only bacterial taxa that occurred in at least two triplicates or when more replicates than triplicates were used >50% of the replicates were considered.

Plant	Leaf	Leaf epi	Leaf endo	Flower	Flower epi	Flower endo	Phylum	Class	Order	Family	Genus	
		x					Actinobacteriota	Actinobacteria	Actinomycetales	Beutenbergiaceae	Serinibacter	# **
x	x	x	x	x	x		Actinobacteriota	Actinobacteria	Actinomycetales	Kineococcaceae	Kineococcus	
x	x	x	x	x	x	x	Actinobacteriota	Actinobacteria	Actinomycetales	Microbacteriaceae	Agrococcus	
x	x	x	x		x		Actinobacteriota	Actinobacteria	Actinomycetales	Microbacteriaceae	Clavibacter	
x	x	x	x	x	x	x	Actinobacteriota	Actinobacteria	Actinomycetales	Microbacteriaceae	Frigoribacterium	
x	x	x	x		x		Actinobacteriota	Actinobacteria	Actinomycetales	Microbacteriaceae	Frondihabitans	
		x			x		Actinobacteriota	Actinobacteria	Actinomycetales	Microbacteriaceae	Plantibacter	
		x					Actinobacteriota	Actinobacteria	Actinomycetales	Microbacteriaceae	Pseudoclavibacter	#
x	x	x	x		x		Actinobacteriota	Actinobacteria	Mycobacteriales	Geodermatophilaceae	Klenkia	
	x	x	x				Actinobacteriota	Actinobacteria	Mycobacteriales	Mycobacteriaceae	Aldersonia	
x	x		x	x		x	Actinobacteriota	Actinobacteria	Propionibacteriales	Propionibacteriaceae	Cutibacterium	**
	x	x	x				Bacteroidota	Bacteroidia	Cytophagales	Hymenobacteraceae	Hymenobacter	**
	x	x	x				Bacteroidota	Bacteroidia	Cytophagales	Spirosomaceae	Spirosoma	
		x					Cloacimonadota	Cloacimonadia	Cloacimonadales	Cloacimonadaceae	UBA1060	#
						x	Cyanobacteria	Cyanobacteriia	Pseudanabaenales	Pseudanabaenaceae	Pseudanabaena	# **
x		x	x		x		Deinococcota	Deinococci	Deinococcales	Deinococcaceae	Deinococcus	
		x					Deinococcota	Deinococci	Deinococcales	Trueperaceae	MPNL01	#
		x					Deinococcota	Deinococci	Deinococcales	Trueperaceae	Truepera	#
		x					Firmicutes	Bacilli	Haloplasmatales	Turicibacteraceae	Turicibacter	#
					x		Firmicutes	Bacilli	Lactobacillales	Enterococcaceae	Enterococcus	#
x	x	x	x	x	x	x	Firmicutes	Bacilli	Lactobacillales	Lactobacillaceae	Lactobacillus	
					x		Firmicutes	Bacilli	Lactobacillales	Lactobacillaceae	Leuconostoc	# **
		x					Firmicutes	Bacilli	Lactobacillales	Lactobacillaceae	Pediococcus	#
		x					Firmicutes	Clostridia	Peptostreptococcales	Peptostreptococcaceae	GCA-900066495	#
	x	x			x		Proteobacteria	Alphaproteobacteria	Acetobacterales	Acetobacteraceae	Acetobacter	**
x		x			x		Proteobacteria	Alphaproteobacteria	Acetobacterales	Acetobacteraceae	Asaia	
		x					Proteobacteria	Alphaproteobacteria	Caedimonadales	Caedimonadaceae	Caedimonas	# **
		x					Proteobacteria	Alphaproteobacteria	Rhizobiales	Pleomorphomonadaceae	Pleomorphomonas	#
x	x	x	x	x	x	x	Proteobacteria	Alphaproteobacteria	Rhizobiales	Rhizobiaceae	Leaf454	
				x	x	x	Proteobacteria	Alphaproteobacteria	Rickettsiales	Anaplasmataceae	Wolbachia	
x	x	x	x	x	x	x	Proteobacteria	Alphaproteobacteria	Rickettsiales	Rickettsiaceae	Rickettsia	
		x					Proteobacteria	Alphaproteobacteria	Sphingomonadales	Sphingomonadaceae	B12	#
	x	x	x				Proteobacteria	Alphaproteobacteria	Sphingomonadales	Sphingomonadaceae	FW-11	
	x	x					Proteobacteria	Gammaproteobacteria	Burkholderiales	Burkholderiaceae	Comamonas	
		x					Proteobacteria	Gammaproteobacteria	Burkholderiales	Burkholderiaceae	Curvibacter	#
						x	Proteobacteria	Gammaproteobacteria	Burkholderiales	Burkholderiaceae	Ralstonia	#
	x	x					Proteobacteria	Gammaproteobacteria	Burkholderiales	Burkholderiaceae	Rugamonas	
					x		Proteobacteria	Gammaproteobacteria	Enterobacterales	Enterobacteriaceae	Atlantibacter	# **
x	x	x	x	x	x	x	Proteobacteria	Gammaproteobacteria	Enterobacterales	Enterobacteriaceae	Buttiauxella	
					x		Proteobacteria	Gammaproteobacteria	Enterobacterales	Enterobacteriaceae	Cedecea	# **
		x		x	x	x	Proteobacteria	Gammaproteobacteria	Enterobacterales	Enterobacteriaceae	Citrobacter	
				x	x		Proteobacteria	Gammaproteobacteria	Enterobacterales	Enterobacteriaceae	Enterobacter	
x	x	x		x	x	x	Proteobacteria	Gammaproteobacteria	Enterobacterales	Enterobacteriaceae	Erwinia	
				x	x	x	Proteobacteria	Gammaproteobacteria	Enterobacterales	Enterobacteriaceae	Escherichia	
					x		Proteobacteria	Gammaproteobacteria	Enterobacterales	Enterobacteriaceae	Gilliamella	#
				x		x	Proteobacteria	Gammaproteobacteria	Enterobacterales	Enterobacteriaceae	Nissabacter	
				x	x		Proteobacteria	Gammaproteobacteria	Enterobacterales	Enterobacteriaceae	Pectobacterium	
						x	Proteobacteria	Gammaproteobacteria	Enterobacterales	Enterobacteriaceae	Providencia	# **
				x	x	x	Proteobacteria	Gammaproteobacteria	Enterobacterales	Enterobacteriaceae	Rosenbergiella	
				x	x		Proteobacteria	Gammaproteobacteria	Enterobacterales	Enterobacteriaceae	Serratia	
						x	Proteobacteria	Gammaproteobacteria	Enterobacterales	Enterobacteriaceae	SoCistrobi	#
						x	Proteobacteria	Gammaproteobacteria	Enterobacterales	Enterobacteriaceae	Sodalis	#
					x		Proteobacteria	Gammaproteobacteria	Enterobacterales	Enterobacteriaceae	Tatumella	#
					x		Proteobacteria	Gammaproteobacteria	Pseudomonadales	Moraxellaceae	Acinetobacter	#

Legend:
** never detected in soil – exclusive to *Achillea millefolium*
# plant compartment specific core-taxa

TABLE S4 I Taxonomy of core fungi of the total plant, total leaf, epi- and endophytic leaf and flower communities of *Achillea* not detected in the soil core microbiome. Occurrence in different plant compartments is marked with x. To determine core and specific plant tissue community, only fungal taxa that occurred in at least two triplicates or when more replicates than triplicates were used >50% of the replicates were considered.

Plant	Leaf	Leaf epi	Leaf endo	Flower	Flower epi	Flower endo	Phylum	Class	Order	Family	Genus	
				x		x	Ascomycota	Dothideomycetes	Botryosphaeriales	Botryosphaeriaceae	Botryosphaeria	
		x					Ascomycota	Dothideomycetes	Capnodiales	Cladosporiaceae	Rachicladosporium	# **
				x	x	x	Ascomycota	Dothideomycetes	Capnodiales	Dissoconiaceae	Dissoconium	
x	x	x	x	x	x	x	Ascomycota	Dothideomycetes	Capnodiales	Mycosphaerellaceae	Ramularia	
		x					Ascomycota	Dothideomycetes	Capnodiales	Mycosphaerellaceae	Septoria	#
	x	x	x				Ascomycota	Dothideomycetes	Capnodiales	Mycosphaerellaceae	Sphaerulina	
		x					Ascomycota	Dothideomycetes	Capnodiales	Mycosphaerellaceae	Zymoseptoria	# **
x	x	x	x	x	x	x	Ascomycota	Dothideomycetes	Dothideales	Aureobasidiaceae	Aureobasidium	
x	x	x	x	x	x		Ascomycota	Dothideomycetes	Pleosporales	Didymellaceae	Ascochyta	
x	x	x	x	x	x	x	Ascomycota	Dothideomycetes	Pleosporales	Didymellaceae	Neoascochyta	
	x		x				Ascomycota	Dothideomycetes	Pleosporales	Didymellaceae	Stagonosporopsis	
			x				Ascomycota	Dothideomycetes	Pleosporales	Leptosphaeriaceae	Leptosphaeria	#
		x					Ascomycota	Dothideomycetes	Pleosporales	Massarinaceae	Massarina	#
	x	x	x				Ascomycota	Dothideomycetes	Pleosporales	Massarinaceae	Stagonospora	
	x		x		x		Ascomycota	Dothideomycetes	Pleosporales	Periconiaceae	Periconia	
		x					Ascomycota	Dothideomycetes	Pleosporales	Phaeosphaeriaceae	Chaetosphaeronema	#
	x	x	x				Ascomycota	Dothideomycetes	Pleosporales	Phaeosphaeriaceae	Leptospora	
x	x	x	x				Ascomycota	Dothideomycetes	Pleosporales	Phaeosphaeriaceae	Phaeosphaeria	
			x				Ascomycota	Dothideomycetes	Pleosporales	Phaeosphaeriaceae	Pseudoophiobolus	# **
	x		x				Ascomycota	Dothideomycetes	Pleosporales	Phaeosphaeriaceae	Septoriella	**
x	x	x	x		x		Ascomycota	Dothideomycetes	Pleosporales	Phaeosphaeriaceae	unidentified Phaeosphaeriaceae	
x	x	x	x	x	x	x	Ascomycota	Dothideomycetes	Pleosporales	Pleosporaceae	Alternaria	
x	x			x	x		Ascomycota	Dothideomycetes	Pleosporales	Pleosporaceae	Stemphylium	**
		x					Ascomycota	Dothideomycetes	Pleosporales	Pleosporaceae	unidentified Pleosporaceae	#
	x		x				Ascomycota	Leotiomycetes	Helotiales	Helotiaceae	Crocicreas	
		x					Ascomycota	Leotiomycetes	Helotiales	Helotiales	Mycochaetophora	#
x	x	x		x	x	x	Ascomycota	Leotiomycetes	Helotiales	Sclerotiniaceae	Botrytis	
		x					Ascomycota	Leotiomycetes	Thelebolales	Pseudeurotiaceae	unidentified Pseudeurotiaceae	# **
			x				Ascomycota	Taphrinomycetes	Taphrinales	Protomycetaceae	Protomyces	#
		x					Ascomycota	Taphrinomycetes	Taphrinales	Taphrinaceae	Taphrina	# **
					x		Basidiomycota	Agaricomycetes	Russulales	Peniophoraceae	Peniophora	#
			x				Basidiomycota	Cystobasidiomycetes	Cystobasidiomycetes	Buckleyzymaceae	Buckleyzyma	#
	x	x	x				Basidiomycota	Exobasidiomycetes	Entylomatales	Entylomatales	Tilletiopsis	
	x	x	x				Basidiomycota	Microbotryomycetes	Leucosporidiales	Leucosporidiaceae	Leucosporidium	
x	x	x	x	x	x	x	Basidiomycota	Microbotryomycetes	Sporidiobolales	Sporidiobolaceae	Sporobolomyces	
x	x	x	x	x	x	x	Basidiomycota	Microbotryomycetes	Sporidiobolales	Sporidiobolaceae	unidentified Sporidiobolaceae	**
					x		Basidiomycota	Pucciniomycetes	Pucciniales	Pucciniaceae	Puccinia	#
	x	x	x				Basidiomycota	Tremellomycetes	Cystofilobasidiales	Mrakiaceae	Itersonilia	
			x				Basidiomycota	Tremellomycetes	Cystofilobasidiales	Mrakiaceae	Udeniomyces	# **
x	x	x	x	x	x	x	Basidiomycota	Tremellomycetes	Filobasidiales	Filobasidiaceae	Filobasidium	
	x	x	x				Basidiomycota	Tremellomycetes	Holtermanniales	Holtermanniales	Holtermanniella	
x	x	x	x	x	x	x	Basidiomycota	Tremellomycetes	Tremellales	Bulleraceae	Bullera	
x	x	x	x		x		Basidiomycota	Tremellomycetes	Tremellales	Bulleribasidiaceae	Dioszegia	
					x		Basidiomycota	Tremellomycetes	Tremellales	Tremellaceae	Bulleromyces	#
	x	x	x				Basidiomycota	Tremellomycetes	Tremellales	unidentified Tremellales	unidentified Tremellales	

Legend:
** never detected in soil – exclusive to *Achillea millefolium*
# plant compartment specific core-taxa

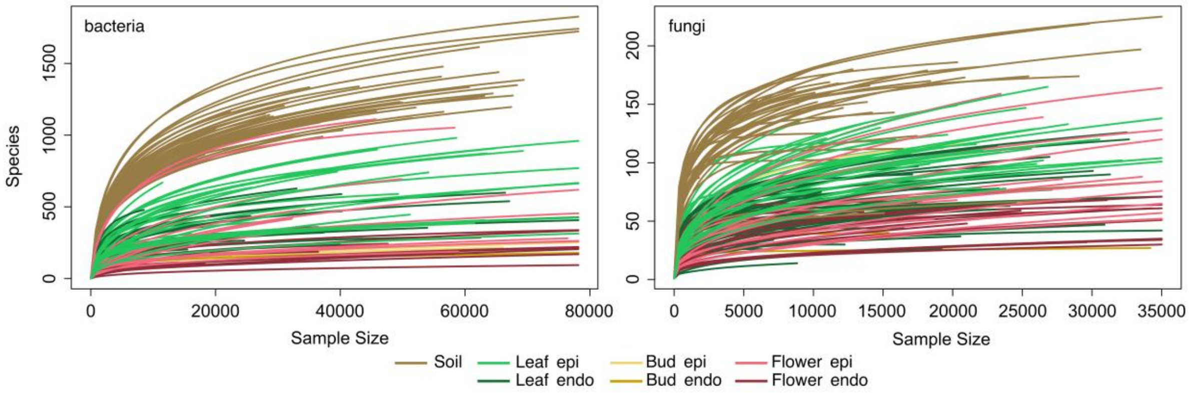

FIGURE S1 l Rarefaction curves of quality-controlled ZOTUs of bacterial species (left) and fungal genera (right) of all samples including soil, leaf, buds and flower of *Achillea* and soil and leaf of *Hamamelis*. The mean coverage per sample of bacterial and fungal species was 72%±11% and 87%±6%, respectively.

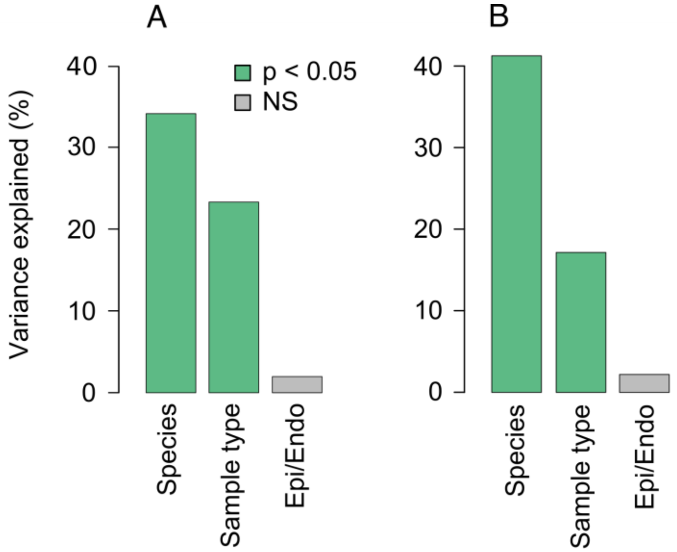

FIGURE S2 l PERMANOVA of all bacterial (A, n=65) and fungal (B, n=69) plant samples of the time-series (soil samples not included) of *Achillea* and *Hamamelis*, including variance explained by plant species, sample type (leaf/flower) and epiphytic/endophytic taxa. NS: not significant.

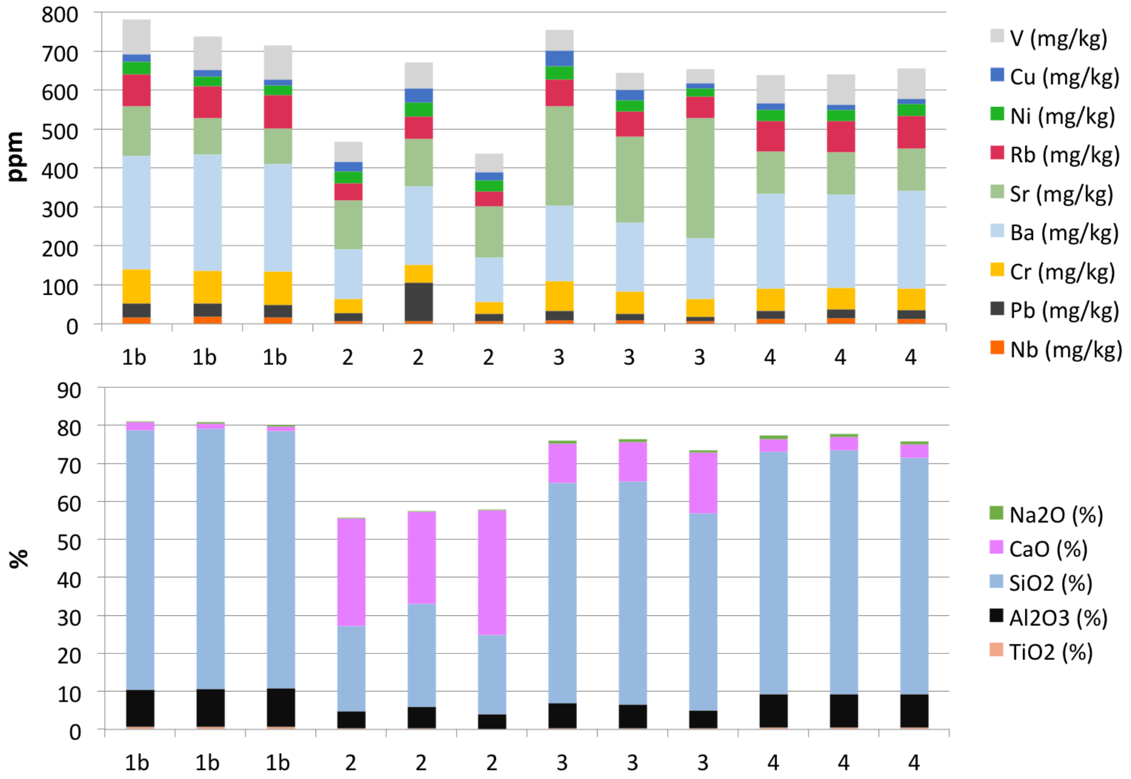

FIGURE S3 I Metal (upper) and metal oxide (lower) constituents of the soil of locations 1 to 4 where *Achillea* samples were collected for the core microbiome assessment. For location details see Table 1. Only elements are listed which were detected at all locations.

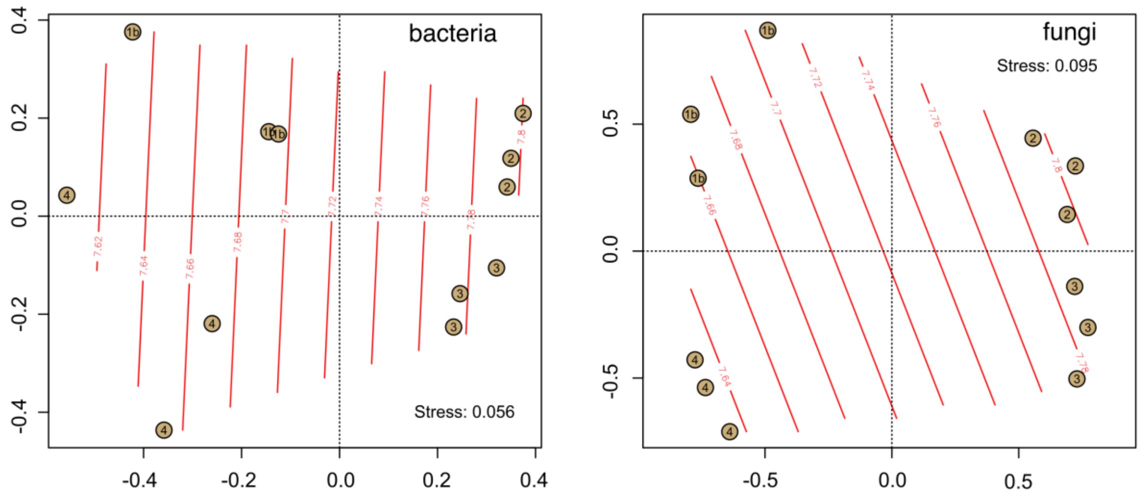

FIGURE S4 I Graph of the NMDS analysis of bacterial (left, n=12) and fungal communities (right, n=12) in the soils of locations 1 to 4 where *Achillea* samples were collected for the core microbiome assessment. Red isoclines indicate pH of the locations. For location details see Table 1. The respective number of location is shown inside the circle.

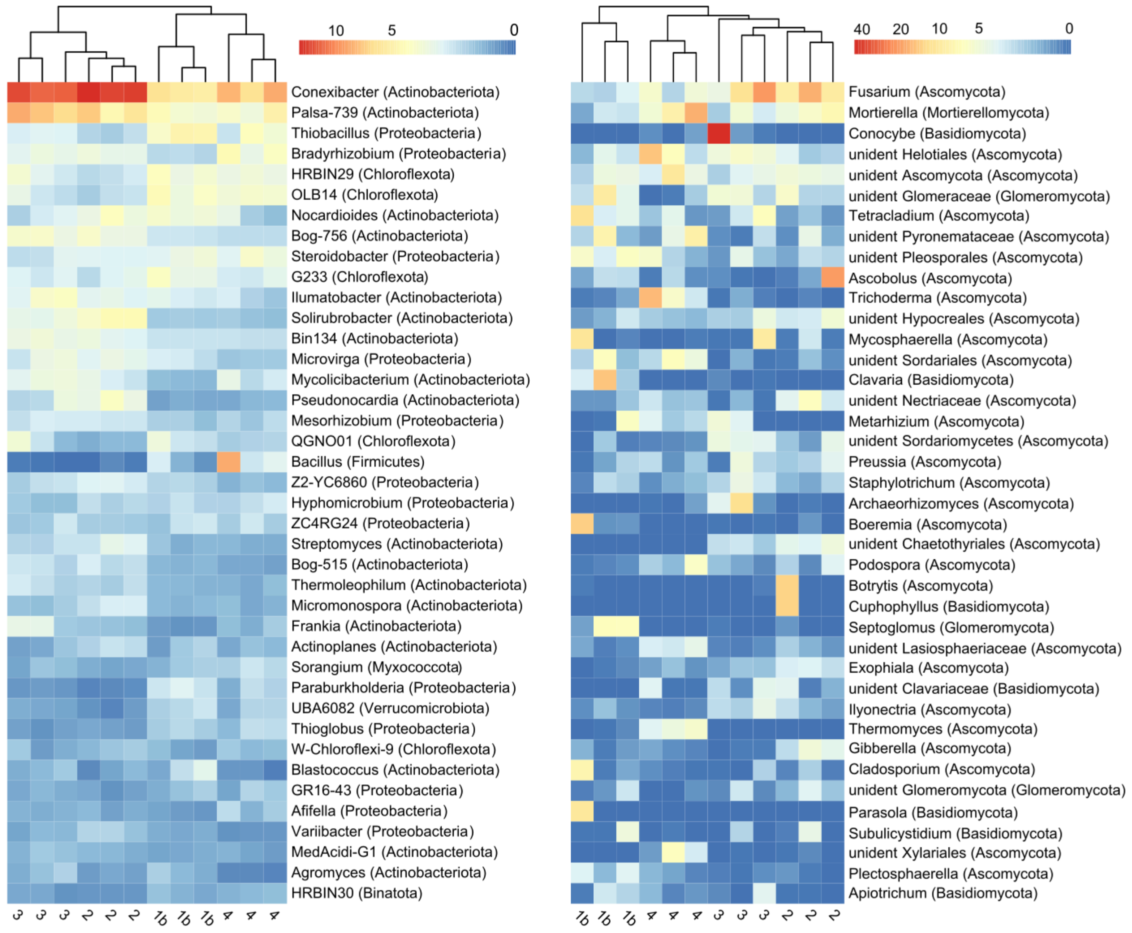

FIGURE S5 I Heat map and cluster analysis of the 40 most abundant bacterial species (left) and fungal genera (right) in the triplicate soil samples at locations 1 to 4, where *Achillea* samples were collected for the core microbiome assessment. Locations are indicated at the bottom and for details see Table 1. Colour code indicates percentages of the abundance of the total community.

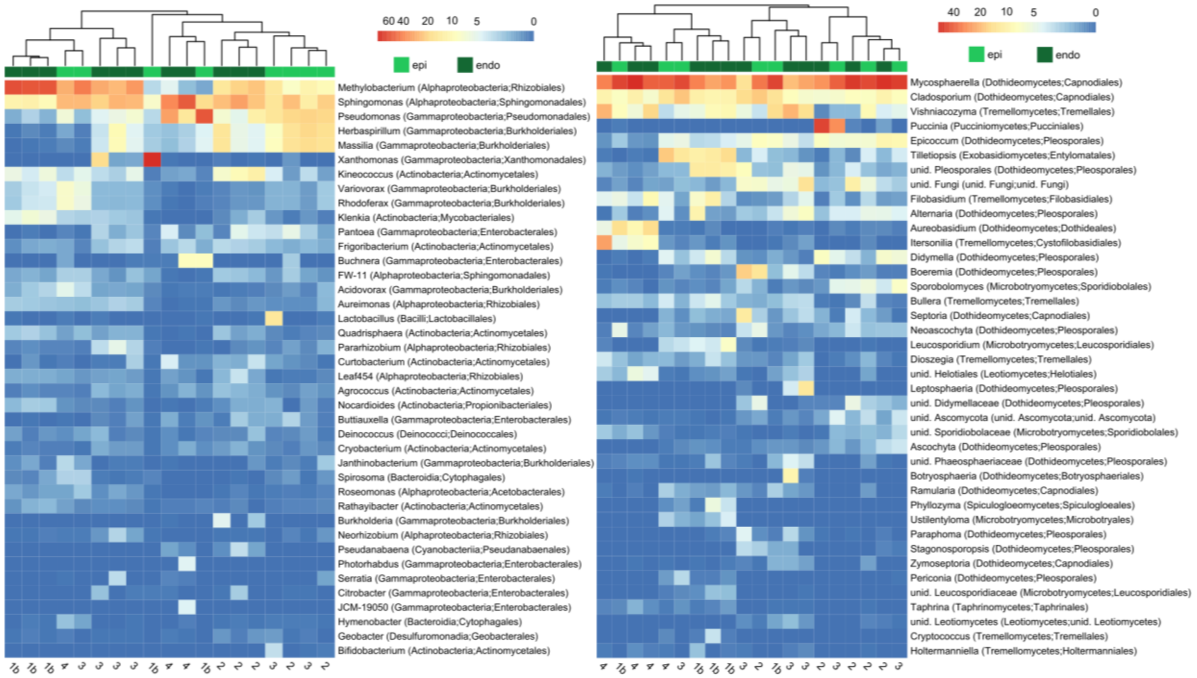

FIGURE S6 I Heat map and cluster analysis of the 40 most abundant bacterial species (left) and fungal genera (right) in the replicate samples of the epi- and endophytic leave compartments of *Achillea* at locations 1 to 4, where samples were collected for the core microbiome assessment. Locations are indicated at the bottom and for details see Table 1. Colour code indicates percentages of the abundance of the total community.

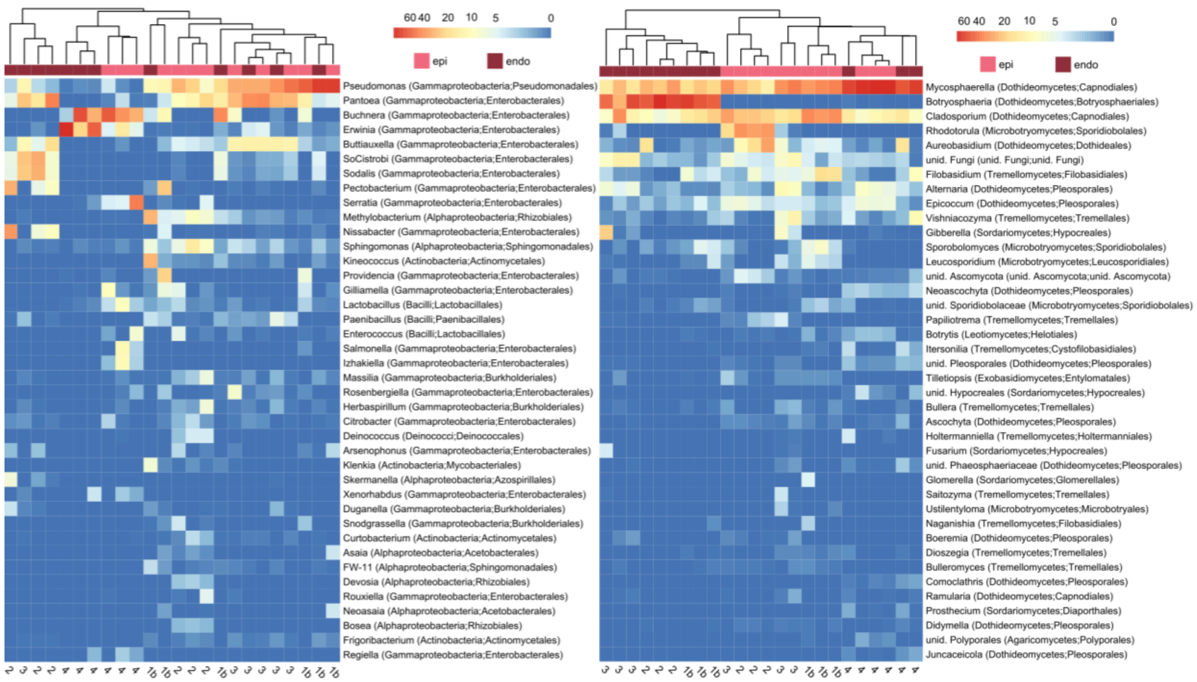

FIGURE S7 I Heat map and cluster analysis of the 40 most abundant bacterial species (left) and fungal genera (right) in the replicate samples of the epi- and endophytic floral compartments of *Achillea* at locations 1 to 4, where samples were collected for the core microbiome assessment. Locations are indicated at the bottom and for details see Table 1. Colour code indicates percentages of the abundance of the total community.

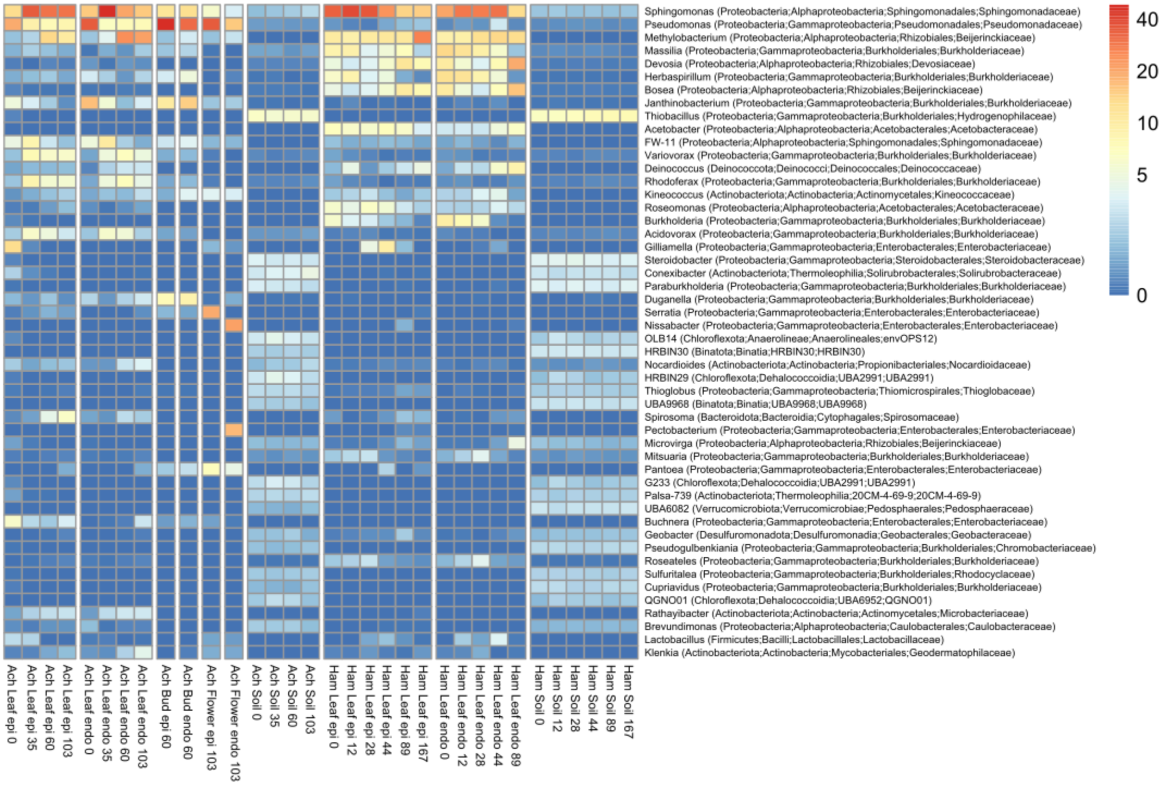

FIGURE S8 I Heat map of the 50 most abundant bacterial genera of the epi- and endophytic leaf, bud and flower and the soil communities of *Achillea* (Ach) over a period of 103 days (April to July) and of the epi- and endophytic leaf and the soil communities of *Hamamelis* (Ham) over periods of 89 and 167 days (April to July and September), respectively. Genus abundance (%) is given as mean values of biological triplicates.

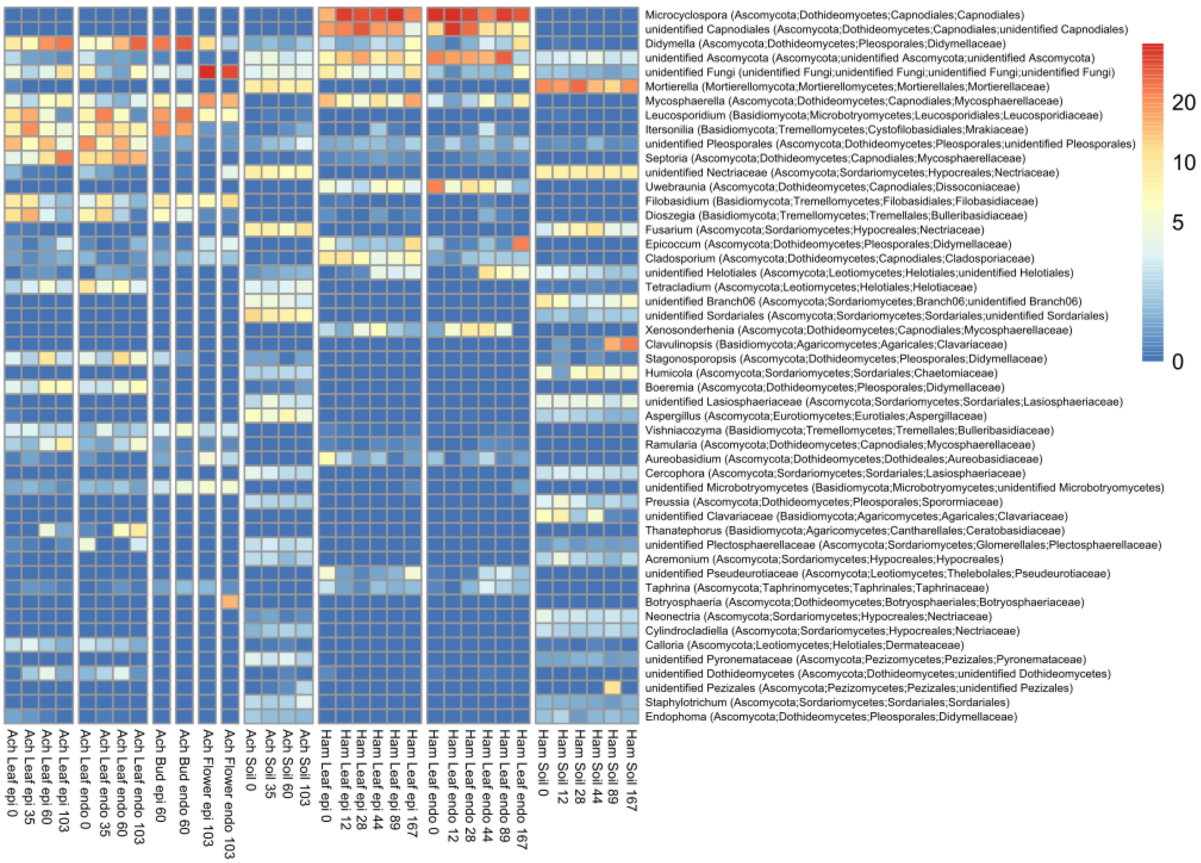

FIGURE S9 l Heat map of the 50 most abundant fungal genera of the epi- and endophytic leaf, bud and flower and the soil communities of *Achillea* (Ach) over a period of 103 days (April to July) and of the epi- and endophytic leaf and the soil communities of *Hamamelis* (Ham) over periods of 89 and 167 days (April to July and September), respectively. Genus abundance (%) is given as mean values of biological triplicates.
